# MAPA: A Semantic Network Framework for Functional Module Discovery and Interpretation in Multi‐Omics Data

**DOI:** 10.1002/advs.77774

**Published:** 2026-09-16

**Authors:** Yifei Ge, Feifan Zhang, Yijiang Liu, Chao Jiang, Peng Gao, Nguan Soon Tan, Sai Zhang, Yuchen Shen, Qianyi Zhou, Xin Zhou, Xiao Wang, Fangqing Zhao, Chuchu Wang, Xiaotao Shen

**Affiliations:** ^1^ Lee Kong Chian School of Medicine Nanyang Technological University Singapore Singapore; ^2^ School of Chemistry Chemical Engineering and Biotechnology Nanyang Technological University Singapore Singapore; ^3^ Life Sciences Institute Zhejiang University Hangzhou Zhejiang China; ^4^ Department of Environmental Health and Department of Molecular Metabolism Harvard T.H. Chan School of Public Health Boston Massachusetts USA; ^5^ School of Biological Sciences Nanyang Technological University Singapore Singapore; ^6^ Department of Biomedical Informatics & Data Science Yale University School of Medicine Yale University New Haven CT USA; ^7^ Centre of Biomedical Systems and Informatics International Campus ZJU‐UoE Institute Zhejiang University School of Medicine Zhejiang University Haining Zhejiang China; ^8^ Intelligent Medicine Institute Fudan Microbiome Center Fudan University Shanghai Medical College Fudan University Shanghai China; ^9^ State Key Laboratory of Crop Stress Adaptation and Improvement State Key Laboratory of Cotton Bio‐Breeding and Integrated Utilization Henan Joint International Laboratory for Crop Multi‐Omics Research School of Life Sciences Henan University Kaifeng Henan China; ^10^ State Key Laboratory of Animal Biodiversity Conservation and Integrated Pest Management Institute of Zoology Chinese Academy of Sciences Beijing China; ^11^ Singapore Phenome Center Nanyang Technological University Singapore Singapore

**Keywords:** biological computation, language model, multi omics, systems biology

## Abstract

Multi‐omics technologies generate high‐dimensional molecular signatures that provide unprecedented opportunities to uncover biological mechanisms. However, translating complex molecular alterations into coherent and interpretable functional insights remains a major challenge. Existing module discovery methods can identify groups of related features, but often lack direct biological interpretability, whereas pathway‐based approaches frequently yield redundant results that complicate interpretation. Here, we present MAPA (Modular Analysis and Phenotype‐informed Annotation using large language models [LLMs]), a semantic‐biological network framework for functional module discovery and interpretation in multi‐omics data. MAPA integrates molecular interactions and pathway‐level functional context into a unified semantic‐biological network, and applies random walk with restart to quantify global functional relatedness among molecules and pathways for coherent module discovery across omics layers. MAPA further incorporates LLM‐assisted interpretation with retrieval‐augmented generation (RAG) to produce structured, literature‐informed module interpretation. Benchmarking against existing approaches shows that MAPA achieves superior module reconstruction and expert‐aligned functional interpretation. Applied to aging‐related multi‐omics datasets, MAPA reveals biologically coherent modules and biological insights that are difficult to obtain from conventional pathway analyses alone. MAPA provides a generalizable framework for organizing fragmented and heterogeneous molecular features into functional modules and comprehensive interpretations.

## Graphical abstract

MAPA transforms complex multi‐omics data into biologically coherent functional modules by integrating pathway information with molecular interaction networks. Retrieval‐augmented large language models then generate structured, literature‐informed interpretations. This unified framework improves module reconstruction and reveals cross‐omics relationships among transcripts, proteins, metabolites and pathways that are difficult to capture with conventional pathway analyses alone.

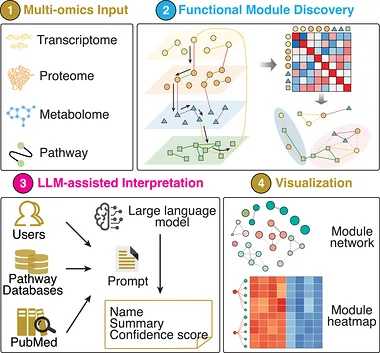

## 1 Introduction

Multi‐omics technologies have transformed biomedical research by enabling comprehensive profiling of molecular alterations across transcripts, proteins, and metabolites [1, 2]. These advances provide an unprecedented opportunity to investigate biological systems across multiple molecular layers and their interactions, and to uncover mechanisms underlying physiological and pathological processes [1, 2, 3]. However, a central challenge remains: how to translate complex molecular signatures into coherent, biologically meaningful insights. Identifying structured and interpretable functional modules from high‐dimensional data has therefore become an important strategy for multi‐omics data interpretation [4, 5, 6, 7].

A wide range of multi‐omics integration methods has been developed to address this challenge [4, 5, 6, 7, 8, 9]. Among them, network‐based approaches are particularly attractive for mechanism‐oriented analysis because they can represent heterogeneous molecules and their interactions within a shared biological framework [10, 11]. For example, knowledge‐guided molecular networks can connect genes, proteins, and metabolites through curated interactions and thereby support module discovery and interpretation [12, 13]. However, some approaches directly identify modules as densely connected local subnetworks [12]. As a result, they may overlook molecules that participate in the same functional theme but are linked mainly through indirect relationships rather than obvious topological proximity [6]. To address this limitation, network propagation was developed to quantify functional relatedness from the global structure of the network [10]. In particular, the random walk with restart (RWR) diffuses information through the network and yields a steady‐state distribution, referred to here as a diffusion profile, that reflects the global proximity of nodes within the system [14, 15]. Although RWR has often been used for candidate prioritization or local subnetwork extraction [6, 16], the resulting diffusion profile can also be interpreted as a representation of how the influence of a node of interest propagates across the network [15]. Therefore, comparing these profiles provides a natural basis for quantifying functional similarity between nodes from their global propagation patterns.

An alternative and widely adopted strategy for interpreting multi‐omics data is pathway analysis, in which differential molecules are mapped onto curated biological pathways to identify pathways associated with the phenotype [17, 18]. By leveraging prior knowledge, this strategy provides an interpretable framework for contextualizing molecular signatures [19]. However, enriched pathways are usually treated as downstream interpretations rather than as structured components of the network [12]. Even when enriched pathways are incorporated as an additional network layer, functionally related pathways remain disconnected [6]. Integrating pathway similarity [20, 21] into the network can therefore extend the functional landscape, allowing related pathways and their associated molecules to be connected even in the absence of direct molecular interaction edges.

Even when coherent multi‐omics modules are identified, their biological interpretation remains labor‐intensive and difficult to standardize [17]. This challenge is particularly pronounced when modules contain heterogeneous molecules and pathways, as supporting evidence is often fragmented across studies and continues to expand rapidly in the biomedical literature [22]. Recent advances in large language models (LLMs) offer a practical route for standardizing module‐level interpretation, because LLMs can summarize heterogeneous biological information and synthesize evidence distributed across pathway databases and the literature [23, 24, 25]. When coupled with retrieval‐augmented generation (RAG), LLMs can dynamically incorporate literature evidence, improving factual grounding while mitigating hallucinations [26]. This provides a scalable approach for comprehensive module‐level interpretation [24].

More fundamentally, existing approaches lack a unified computational representation of biological function. As a result, functional module discovery and biological interpretation remain fragmented processes, often requiring extensive manual synthesis by domain experts. This limitation becomes particularly pronounced in multi‐omics studies, where transcripts, proteins and metabolites exist in fundamentally different feature spaces and cannot be directly compared using conventional similarity metrics.

Here we introduce MAPA (Modular Analysis and Phenotype‐informed Annotation using LLMs), a network‐based framework for functional module discovery and interpretation in multi‐omics data. MAPA builds on established text‐embedding‐based pathway similarity [27] and random walk with restart on multiplex‐heterogeneous networks [14]. Its principal contribution is to integrate pathway semantic relationships, curated molecular interactions, diffusion‐profile‐based cross‐omics module discovery, and retrieval‐augmented LLM interpretation within a unified workflow. MAPA constructs a knowledge‐guided multi‐layer network and derives diffusion profiles for molecules and pathways, which are used as global functional representations for systematic similarity quantification and module discovery. It then applies an LLM with RAG to generate structured, literature‐informed interpretations of the identified modules. Through benchmarking and applications to multi‐omics aging datasets, we show that MAPA reconstructs coherent functional modules and facilitates biologically meaningful interpretation that is less accessible with conventional approaches.

## 2 Results

### 2.1 MAPA Enables Semantic Discovery and Interpretation of Functional Modules Across Multi‐Omics Layers

**FIGURE 1:**
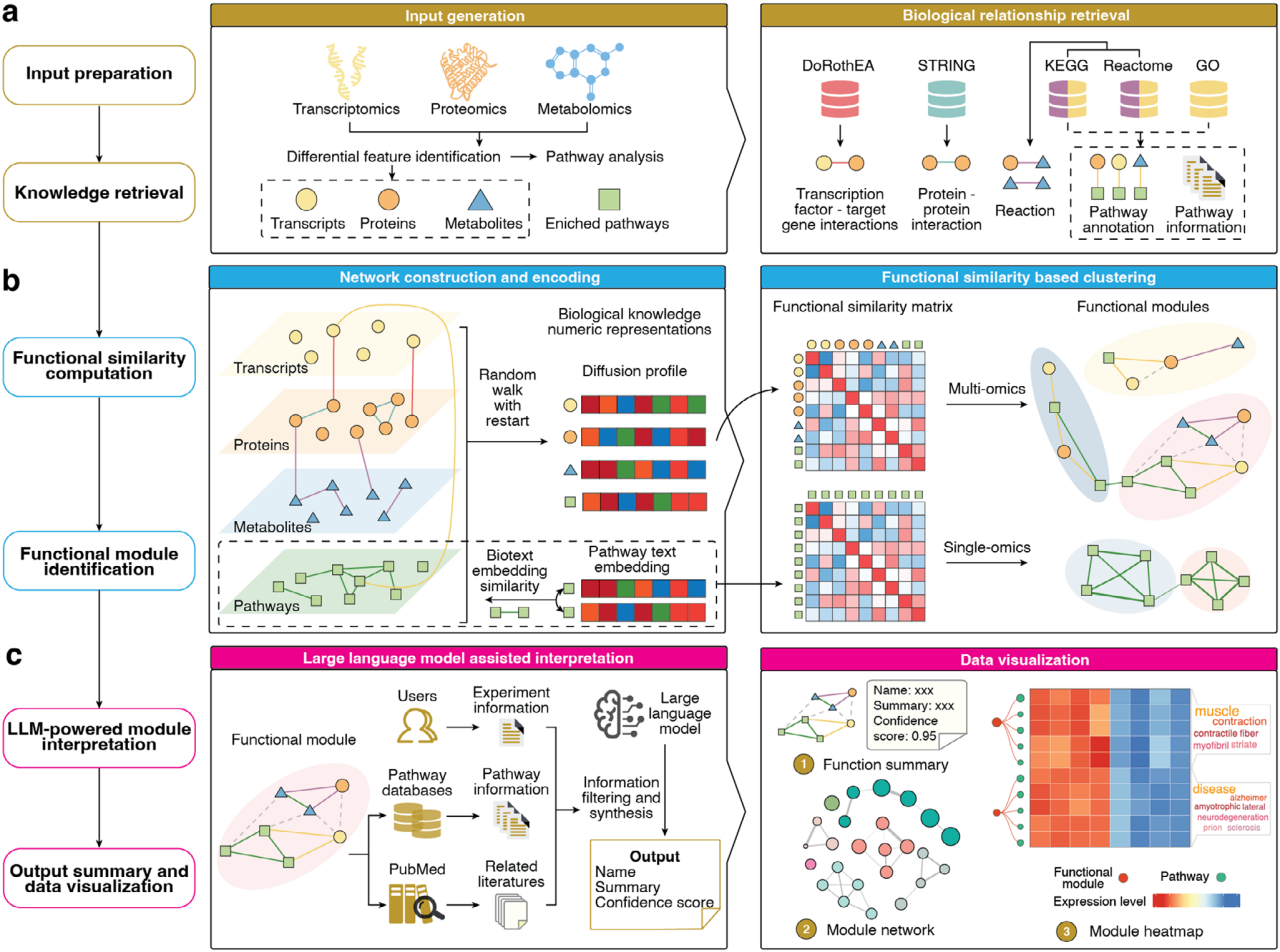
Overview of the MAPA framework. (a) MAPA takes transcriptomic, proteomic, and metabolomic features as input and performs pathway enrichment analysis to obtain pathway‐level functional information. (b) MAPA constructs a network connecting molecules and pathways and discovers the functional modules. (c) MAPA generates LLM‐assisted function interpretation using module components, experimental information, and related literature, and provides multiple visualizations to support module interpretation.

MAPA consists of three major stages: (1) input preparation and knowledge retrieval, (2) semantic‐biological similarity quantification and functional module discovery, and (3) retrieval‐augmented LLM‐assisted module interpretation and visualization (Figure 1a‐c).

In the first stage, MAPA prepares the molecular and pathway‐level inputs for downstream semantic integration (Figure 1a and Methods). The input consists of phenotype‐associated molecules, such as differentially expressed transcripts, proteins, or metabolites. Pathway enrichment analysis, using either over‐representation analysis (ORA) [28] or gene set enrichment analysis (GSEA) [29], is subsequently performed to identify enriched pathways. MAPA then retrieves curated molecular relationships from established biological knowledge resources, including transcription factor‐target interactions [30], protein‐protein interactions [31], reaction relationships [32, 33], and pathway/gene‐ontology (GO) annotation [32, 33, 34, 35]. In parallel, pathway and GO term descriptions are transformed into high‐dimensional semantic embeddings using a text embedding model (Methods). Pairwise functional similarity between pathways is quantified by cosine similarity between these embeddings, which we term biotext embedding similarity. This enables quantitative comparison of biological functions across heterogeneous pathway databases within a shared semantic space [27].

In the second stage, MAPA integrates molecular interactions, pathway annotations, and semantic pathway relationships into a unified heterogeneous network to model functional relationships across omics layers (Figure 1b and Methods). In the multi‐omics setting, all molecules and enriched pathways are represented as nodes in the same network. Molecular nodes are connected by curated relationships, pathway nodes are connected by biotext embedding similarity, and molecule‐pathway edges are defined by pathway annotations (Methods). This design integrates molecular interaction knowledge with pathway‐level functional context, allowing molecules from different omics layers to be compared within a shared biological network. We use the term semantic‐biological network to refer to an integrated network in which curated molecular relationships are combined with text‐derived semantic similarities between biological pathways. To quantify functional similarity among molecules and pathways, MAPA applies random walk with restart (RWR) on the integrated network to model how a functional signal initiated from each node propagates through curated molecular/pathway relationships [14]. For each molecule or pathway, MAPA computes a diffusion profile, which records how strongly the signal from that node reaches all other nodes through network propagation (Methods). This diffusion profile serves as a network‐based functional fingerprint of the node and represents its broader functional context [15]. Similarity between diffusion profiles is then used to quantify functional relatedness between all nodes, enabling molecules and pathways from different omics layers to be compared within a shared biological network space. Clustering algorithms are subsequently applied to the diffusion‐profile similarity matrix to discover functional modules (Methods). In the multi‐omics setting, each module may contain molecules and pathways spanning different omics layers, whereas in the single‐omics setting, MAPA discovers modules as groups of functionally coherent pathways (Figure 1b). Unlike local connectivity‐based module detection, diffusion profiles enable MAPA to capture higher‐order functional relationships mediated through indirect biological and semantic connections.

In the third stage, MAPA interprets the identified functional modules using a retrieval‐augmented LLM workflow and generates interpretable outputs (Figure 1c, Figure S1, and Methods). For each module, MAPA uses module‐associated molecules, pathways, and optional experimental context to retrieve relevant literature from PubMed. Retrieved abstracts are first ranked by semantic similarity to the module information and then further prioritized using LLM‐generated relevance scores (Figure S1a). The selected literature, together with module composition and experimental information, is incorporated into a structured prompt to guide LLM‐assisted interpretation. The resulting module interpretation includes a concise module name, a functional summary, and an LLM‐derived confidence score representing a heuristic assessment of the support for a coherent biological interpretation (Figure S1b). To support interpretation and reproducibility, MAPA also generates visualization outputs and reports, including module networks, pathway‐molecule relationships, expression heatmaps, and key metadata for the analysis (Figures S2‐S3).

Together, MAPA establishes a semantic‐biological framework for transforming fragmented multi‐omics signals into coherent and mechanistically interpretable functional modules. By integrating semantic pathway representations, curated biological knowledge, diffusion profile‐based network encoding and retrieval‐augmented LLM reasoning, MAPA provides a scalable and interpretable framework for functional module discovery and biological interpretation in both single‐ and multi‐omics settings.

### 2.2 Biotext Embeddings Capture Functional Relationships Across Heterogeneous Pathway Databases

A central challenge in functional module discovery is to quantitatively represent biological relationships between pathways across heterogeneous knowledge resources (Figure 1b). Existing pathway similarity metrics capture only limited aspects of biological relatedness (Supplementary Notes S1‐S2). Component‐based metrics depend on shared annotated genes [36], whereas ontology‐based metrics are largely restricted to GO terms and may not support comparisons across different pathway databases [37]. To address these limitations, we adopted a text‐embedding‐based approach to quantify pathway functional relatedness, which we term biotext embedding similarity (Methods). This approach enables functional similarity estimation both within and across heterogeneous pathway databases.

**FIGURE 2:**
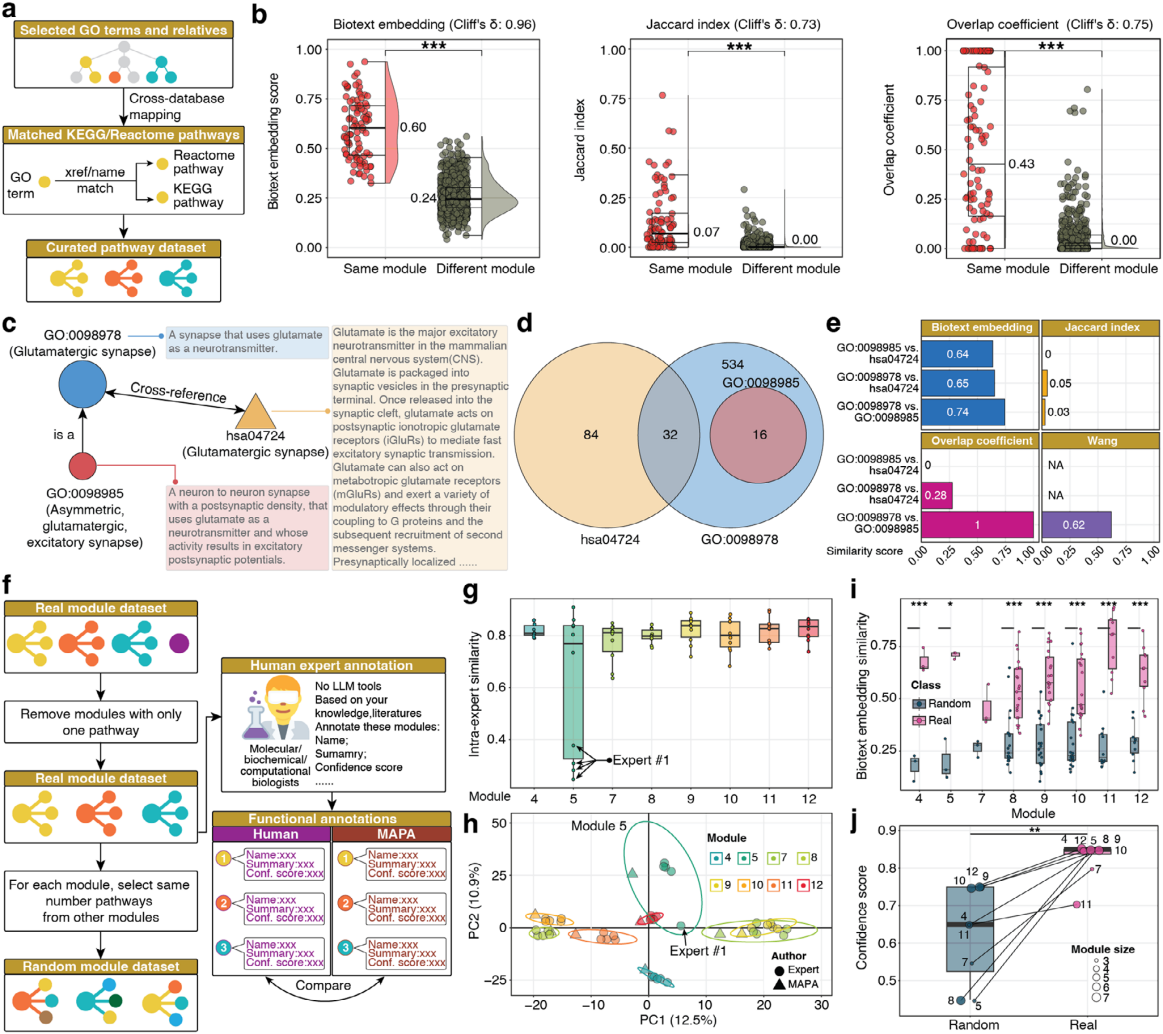
Evaluation of MAPA's core methodological components. (a) Workflow for constructing the curated pathway dataset. (b) Pairwise within‐module (n = 92) and between‐module (n = 854) pathway similarities calculated using biotext embedding, Jaccard index, and overlap coefficient. (c) Case study of one representative module containing three functionally related entries. (d) Gene set overlap among the three entries shown in c. (e) Pairwise similarity among the three entries calculated using biotext embedding, Jaccard index, overlap coefficient, and Wang semantic similarity, where applicable. (f) Workflow for evaluating MAPA‐generated functional interpretation using human expert annotations and size‐matched random modules as negative controls. (g) Pairwise inter‐expert similarities of functional interpretations for each module (n = 10 comparisons per module). (h) Principal‐component analysis (PCA) of module interpretation generated by human experts (circles) and MAPA (triangles). (i) Comparison of intra‐module biotext embedding similarity between real and size‐matched random modules, based on all pathway pairs within each module (n = 3 for Modules 4, 5, and 7; n = 21 for Modules 8–10; n = 10 for Modules 11 and 12, per group). (j) Comparison of MAPA confidence scores for the same real and random modules (n = 8 per group). For b, g, i, and j, center lines indicate medians, boxes indicate the interquartile range (IQR), and whiskers extend to the most extreme values within 1.5 × IQR. For b, i, and j, statistical significance was assessed using Wilcoxon rank‐sum tests. *** *p*‐value < 0.001, ** *p*‐value < 0.01, * *p*‐value < 0.05.

To evaluate this approach, we curated a benchmark dataset containing 44 pathways and GO terms that were manually organized into 12 biologically coherent functional modules (Figure 2a, Supplementary Methods, Figure S4c, and Data S1, Table S1). We initially assessed a broader panel of component‐based metrics, but several showed highly redundant similarity profiles (Figure S5 and Supplementary Data S1, Table S2). Therefore, we selected the Jaccard index and overlap coefficient as representative metrics for subsequent comparison (Figure 2b). Biotext embedding similarity showed a clear separation between within‐module and between‐module pathway pairs, with a larger effect size (Cliff's δ = 0.96) than the Jaccard index (δ = 0.73) and overlap coefficient (δ = 0.75) (Figure 2b). These results indicate that biotext embeddings more effectively capture biologically coherent functional relationships than component‐based metrics.

We next evaluated ontology‐based similarity metrics specifically designed for GO terms. Multiple ontology‐based metrics showed highly consistent pairwise similarity profiles (Figure S6a and Data S1, Table S3), and Wang similarity was selected as a representative approach for comparison (Supplementary Figure S6b). Biotext embedding similarity achieved complete separation between within‐module and between‐module GO‐term pairs, comparable to Wang similarity (Cliff's δ = 1.0 for both methods). However, unlike ontology‐based methods, biotext embedding similarity is not constrained by ontology structure and can therefore be applied across pathway databases and GO sub‐ontologies.

A representative glutamatergic synapse module further highlights the ability of biotext embeddings to recover biologically coherent relationships across heterogeneous pathway resources (Figure 2c). This module contained two related GO terms (GO:0098985 and GO:0098978) together with one KEGG pathway (hsa04724). Although the KEGG pathway shared no annotated genes with GO:0098985 (Figure 2d), biotext embedding similarity assigned consistently high similarity scores among all three entries (0.64‐0.74), accurately reflecting their shared biological theme (Figure 2e and Supplementary Note S3). In contrast, component‐based metrics assigned low or zero similarity scores to these biologically related pairs, whereas Wang similarity could only evaluate the GO‐term pair (Figure 2e).

Together, these results demonstrate that biotext embeddings provide a generalized semantic representation of biological function that supports functional relatedness identification across heterogeneous pathway databases.

### 2.3 MAPA Generates Reproducible and Expert‐aligned Interpretations of Functional Modules

Beyond functional module discovery, MAPA aims to generate biologically coherent and reproducible semantic interpretations for each functional module. We therefore separately evaluated MAPA‐generated module summaries and confidence scores using the curated pathway benchmark dataset (Figure 2f and Methods).

We first evaluated whether MAPA‐generated module interpretations were consistent with human expert interpretations (Methods). Five blinded experts independently annotated the curated modules (Supplementary Data S1, Table S4, and Supplementary Note S4). Expert annotations showed strong semantic concordance across all available annotation pairs (median pairwise cosine similarity = 0.82; IQR: 0.78‐0.85), supporting the biological coherence and interpretability of the curated modules (Figure 2g and Supplementary Methods). We next compared MAPA‐generated interpretations with expert interpretations using biotext embedding similarity. Across nearly all modules, MAPA interpretations showed strong semantic concordance with expert interpretations (Figure 2h), indicating that MAPA generates biologically meaningful module interpretations semantically aligned with human expert interpretations.

We next evaluated the reproducibility of MAPA interpretations across 15 independent runs on the same curated pathway dataset using MAPA's default clustering configuration, which was selected as the best‐performing setting in the clustering‐method evaluation (Methods and Supplementary Figure S4a‐b). Functional module discovery was fully stable across runs, yielding the same 14 modules in every execution [adjusted rand index (ARI) [38] = 1]. The curated benchmark contained 12 expected modules; the two additional MAPA‐derived modules arose because hsa04210 (Apoptosis) and hsa00190 (Oxidative phosphorylation) each separated from their expected module and formed a singleton module (Supplementary Figure S4c‐d and Supplementary Note S5). Repeated MAPA runs generated highly consistent interpretations for the same module across independent executions (cosine similarity > 0.90), whereas interpretations from different modules showed substantially lower similarity (Supplementary Figure S7). These results demonstrate that MAPA produces highly reproducible and robust functional interpretations of modules. Similar robustness was observed when the curated benchmark was repeated with an alternative Qwen‐based embedding and language model, showing concordant pathway biotext embedding similarities (Pearson r = 0.82), substantial clustering agreement with the ground truth (ARI = 0.81), comparable agreement with expert annotations, and significantly higher confidence scores for real than for size‐matched random modules (Supplementary Methods, Supplementary Figure S8, and Supplementary Data S1, Tables S9‐S12).

We next examined whether MAPA confidence score was sensitive to differences in the functional coherence of the input modules. To this end, we constructed a negative control dataset by randomly sampling pathways from unrelated modules while preserving module size (Figure 2f, Methods and Supplementary Data S1, Table S5). Real modules exhibited significantly greater intra‐module semantic coherence than randomly constructed modules, as measured by intra‐module biotext embedding similarity (Figure 2i). We subsequently applied the same MAPA interpretation workflow to both real and random modules. Confidence scores were significantly higher for real modules than for random modules (Figure 2j), indicating that this LLM‐derived heuristic was sensitive to differences in the coherence of the input modules in this benchmark.

Together, these results demonstrate that MAPA generates biologically coherent, expert‐aligned, and highly reproducible interpretations for functional modules, with confidence scores that were sensitive to differences in module functional coherence in this benchmark.

### 2.4 MAPA Outperforms Existing Approaches in Functional Module Discovery and Interpretation

To position MAPA within the current landscape of multi‐omics interpretation frameworks, we first compared its analytical design with representative multi‐omics tools, including OmicsNet [39], netOmics [6], OmicsAnalyst [40], and MintTea [5] (Supplementary Table S1). These methods differ substantially in their analytical strategies. Some construct knowledge‐based molecular subnetworks from lists of molecules [39], whereas others infer data‐driven relationships from abundance matrices and define modules using seed proximity [6], correlation structure [40], or phenotype‐associated co‐variation [5]. However, these approaches generally lack a unified semantic representation of biological function and do not provide systematic module‐level interpretation. In contrast, MAPA integrates curated molecular relationships and pathway semantic context into a unified semantic‐biological framework, discovers modules using global diffusion‐based functional representations, and provides structured, retrieval‐augmented interpretation using LLMs. This comparison highlights the distinct conceptual scope of MAPA among current multi‐omics interpretation frameworks.

**FIGURE 3:**
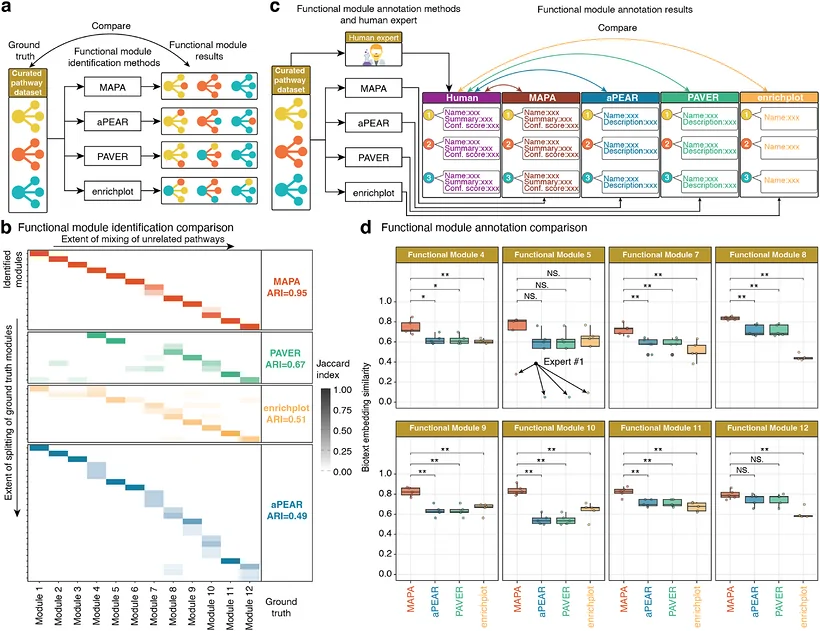
Benchmarking MAPA against existing approaches for functional module discovery and interpretation. (a) Workflow for benchmarking functional module discovery. (b) Comparison of identified modules with ground‐truth modules. Columns indicate ground‐truth modules and rows indicate modules identified by each method. Heatmap values represent the Jaccard index between each identified module and each ground‐truth module. ARI values summarize the overall agreement between identified modules and the ground truth. (c) Workflow for benchmarking functional module interpretation. (d) Comparison of functional module interpretation quality. Box plots show the text embedding cosine similarity between interpretations generated by each method and expert‐curated interpretations across modules. Each box represents five method‐expert annotation comparisons (n = 5). Center lines indicate medians, boxes indicate the IQR, and whiskers extend to the most extreme values within 1.5 × IQR. Statistical significance was assessed using Wilcoxon rank‐sum tests. *** *p*‐value < 0.001; ** *p*‐value < 0.01; * *p*‐value < 0.05; NS., not significant.

We next benchmarked MAPA in a single‐omics setting against widely used tools, including PAVER [27], enrichplot [41], and aPEAR [42] (Methods). These methods differ in how they estimate pathway functional similarity, discover functional modules, and generate module interpretations (Supplementary Table S2). Using the curated pathway benchmark dataset with predefined biologically coherent functional modules (Figure 2a), we first evaluated whether each method could accurately reconstruct the ground‐truth module structure. MAPA achieved near‐complete recovery of the predefined modules, yielding the highest performance with an ARI of 0.95, substantially exceeding PAVER (ARI = 0.67), enrichplot (ARI = 0.51), and aPEAR (ARI = 0.49) (Figure 3b, Supplementary Figure S9, and Supplementary Data S1, Table S6). To further evaluate clustering performance on an independently defined benchmark, we repeated the comparison using the informative GO term dataset (Supplementary Methods, Supplementary Figure S10a and Supplementary Data S2, Tables S1 and S2). MAPA achieved the highest ARI under both default and optimized settings (0.17 and 0.57, respectively), compared with PAVER (0.15 and 0.49), aPEAR (0.02 and 0.13), and enrichplot (−0.001 and 0.11) (Supplementary Figure S10c‐d and Supplementary Data S2, Tables S3 and S6). The lower performance under the default setting may partly reflect the greater overlap in biotext embedding similarity between within‐module and between‐module GO term pairs, making a fixed similarity cutoff less optimal for this benchmark (Supplementary Figure S10b).

We further assessed the ability of each method to generate biologically meaningful module interpretations (Figure 3c and Methods). For each method, module interpretations were compared with expert interpretations using biotext embedding similarity (Supplementary Data S1, Tables S7 and S8). MAPA produced module interpretations with the highest overall semantic similarity to expert interpretations (Figure 3d), indicating closer alignment with expert biological reasoning than existing approaches.

Collectively, these results demonstrate that MAPA more accurately reconstructs biologically coherent functional modules and generates interpretations that more closely align with expert biological reasoning than existing methods.

### 2.5 MAPA Reveals Biologically Coherent and Coordinated Aging‐Associated Functional Modules Across Diverse Tissues

**FIGURE 4:**
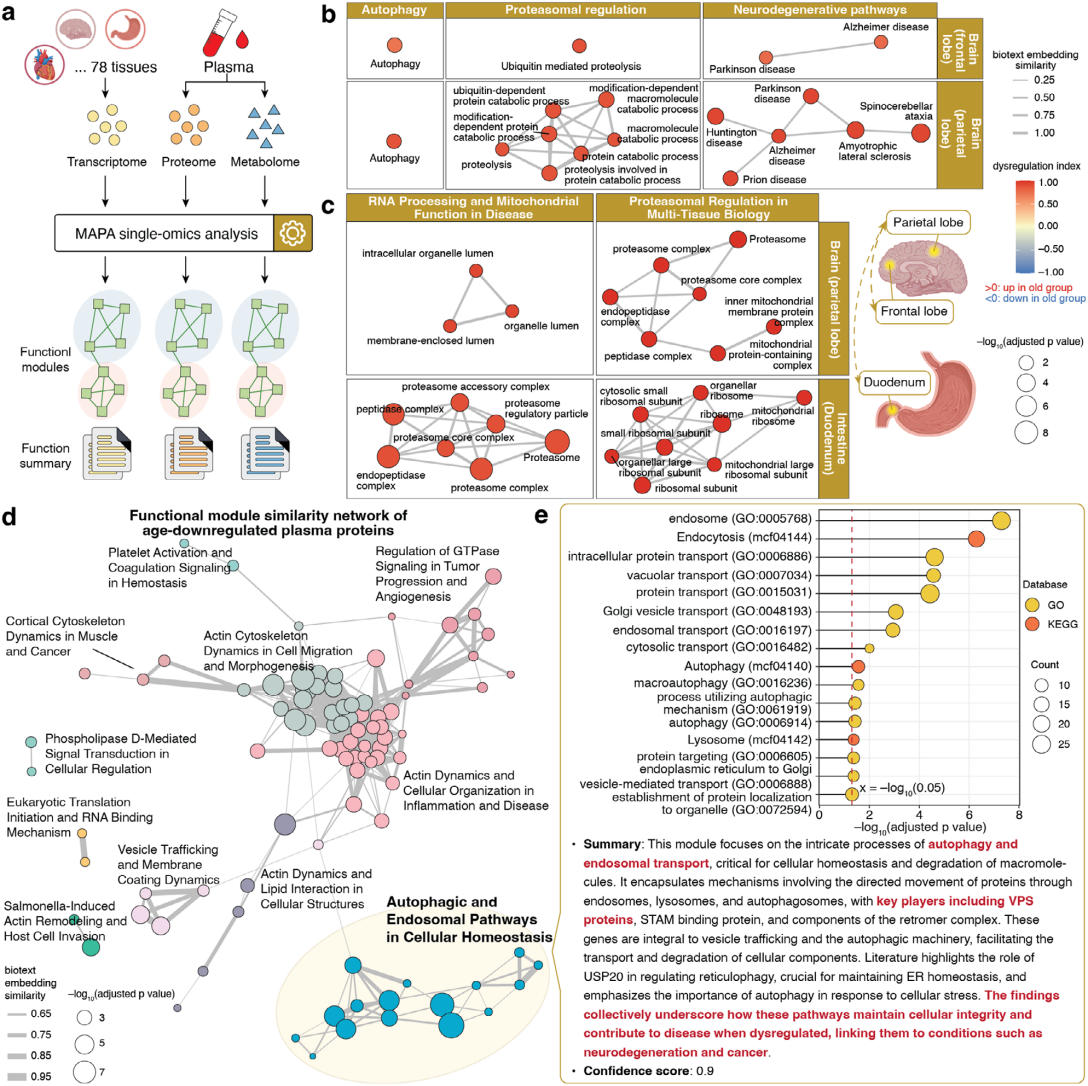
MAPA identifies coordinated aging‐related functional dysregulation across omics datasets. (a) Workflow of the single‐omics case study using MAPA to analyze age‐associated omics datasets. (b) Networks of shared functional modules between the physically adjacent parietal and frontal lobes. (c) Networks of shared functional modules between the parietal lobe and duodenum, highlighting cross‐tissue coordination along the brain‐gut axis. (d) Functional module similarity network of age‐downregulated plasma proteins identified by MAPA. Only modules with more than one pathway are displayed. (e) Representative module related to autophagy and endosomal transport.

To evaluate whether MAPA can recover biologically coherent functional programs from complex omics datasets, we applied it to an aging male cynomolgus monkey dataset from a metformin intervention study, including bulk RNA‐seq‐derived age‐dependent transcriptomic data from 78 tissues together with plasma proteomic and metabolomic data [1] (Figure 4a, Methods, and Supplementary Figure S11 and S12). Age‐associated genes from each tissue were used as input for MAPA analysis. Across the transcriptomic datasets, MAPA identified 162 distinct functional modules across 56 tissues, whereas the remaining 22 tissues showed no significant functional modules (Supplementary Data S3, Tables S1 and S2). To focus on recurrent and biologically interpretable programs, we prioritized modules consistently detected across multiple tissues and calculated a dysregulation index for each tissue‐module pair to summarize the direction and magnitude of aging‐associated changes (Methods, Supplementary Figure S12a, and Supplementary Data S3, Table S3).

MAPA identified coordinated functional changes across anatomically and physiologically related tissues. For example, the frontal and parietal lobes clustered together and showed shared upregulation of modules associated with neurodegenerative pathways, proteasomal regulation and autophagy, indicating coordinated alterations in proteostasis and neurodegeneration‐associated processes during brain aging (Figure 4b). These results indicate that MAPA captures higher‐order functional relationships across related tissues. MAPA also identified coherent functional programs shared across anatomically distant organs. For example, the duodenum and parietal lobe shared upregulation of modules related to proteasomal regulation, RNA processing, and mitochondrial function, suggesting a hypothesis of coordinated dysregulation of protein homeostasis and mitochondrial gene‐expression programs across intestinal and neural tissues (Figure 4c).

Beyond these upregulated programs, MAPA also identified broadly downregulated regulatory processes across tissues. In particular, the module annotated as “FOXO signaling and longevity regulation” was downregulated across multiple tissues, including the lower bronchus, supraspinatus tendon, ileum and liver, suggesting a potential coordinated reduction in longevity‐associated signaling during aging (Supplementary Figure S12b). In contrast, the module annotated as “microRNA dysregulation in cancer progression” exhibited bidirectional regulation, showing downregulation in several peripheral and barrier tissues but upregulation in neural tissues (Supplementary Figure S12a), suggesting tissue‐specific remodeling of microRNA‐mediated regulatory programs during aging.

For the plasma proteomics data, MAPA extended the pathway‐level results from the original study into a more coherent module‐level interpretation (Figure 4d). For example, in the plasma proteomics dataset, the original study identified “Autophagy” as an enriched pathway among age‐downregulated proteins [1]. MAPA further integrated related pathways into a functional module annotated as “Autophagic and Endosomal Pathways in Cellular Homeostasis” through the pathway similarity network (Figure 4d). This module connected autophagy‐related processes with endosomal transport pathways, relationships that were not immediately apparent from individual enrichment results. The corresponding LLM‐assisted interpretation was consistent with established evidence linking impaired autophagy to aging and age‐related diseases [43] (Figure 4e). Notably, the generated interpretation highlighted vacuolar protein sorting proteins as potential mechanistic components connecting autophagy and intracellular trafficking dysfunction, processes implicated in neurodegenerative diseases such as Parkinson's disease [44].

Together, these results demonstrate that MAPA transforms fragmented pathway enrichment outputs into coherent functional programs and biologically interpretable mechanistic hypotheses across tissues and molecular layers.

### 2.6 MAPA Identifies Cross‐Omics Functional Programs Associated with Aging

To evaluate whether MAPA can integrate molecular alterations across omics layers into coherent biological programs, we applied it to a multi‐tissue monkey aging dataset containing transcriptomic, proteomic, and metabolomic profiles across different age groups [3]. We focused on seven tissues that exhibited marked molecular shifts during aging, including the spleen, thymus, aortic arch, ovary, thyroid gland, stomach, and kidney (Methods).

**FIGURE 5:**
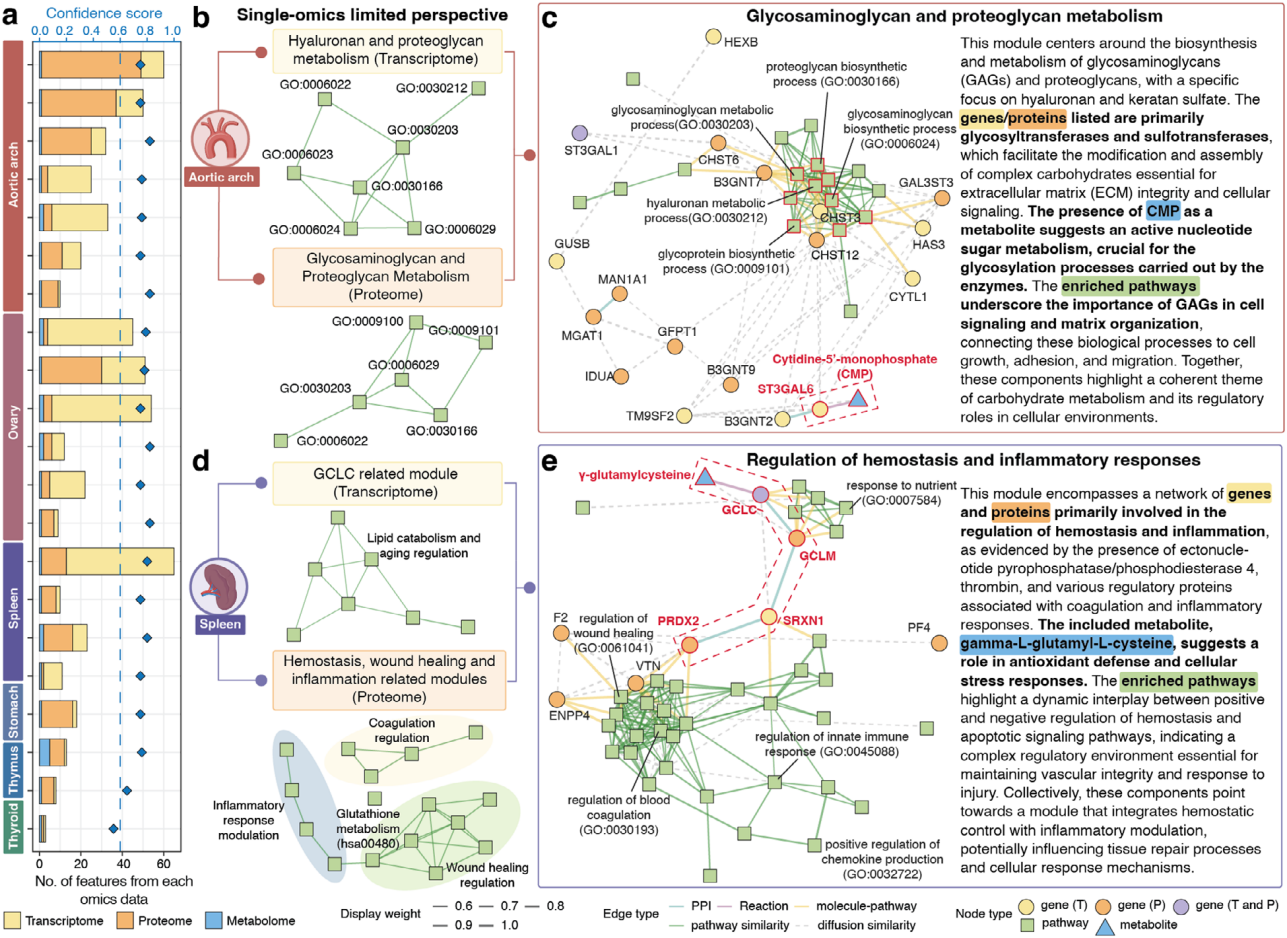
MAPA‐derived multi‐omics functional modules recapitulate known aging signatures and reveal additional cross‐omics connections. (a) Molecular composition of the 21 multi‐omics functional modules across six aging tissues, selected by requiring all three omics types and a module size ≥ 3. Stacked bars show the number of transcriptomic, proteomic, and metabolomic features, and diamonds indicate the LLM‐derived confidence score. (b) Single‐omics analysis identified separate transcriptomic and proteomic modules related to proteoglycan metabolism in the aortic arch. (c) MAPA identified a unified multi‐omics module annotated as glycosaminoglycan and proteoglycan metabolism in the aortic arch. (d) Single‐omics analysis produced a transcriptomic GCLC‐related module and separate proteomic modules associated with hemostasis, wound healing, inflammation, and glutathione metabolism in the spleen. (e) MAPA identified a multi‐omics module annotated as regulation of hemostasis and inflammatory responses, with γ‐glutamylcysteine, GCLC, GCLM, SRXN1, and PRDX2 forming a bridge between the glutathione‐related antioxidant branch and the hemostasis/inflammation branch in the spleen.

Because transcriptomic and proteomic features represent complementary molecular readouts of gene expression, we first assessed metabolite integration using a broader cross‐omics criterion, defined as a module containing at least one metabolite together with at least one transcript or protein. Across the seven tissues, 87 of 373 input metabolites (23.3%) were assigned to 49 metabolite‐containing cross‐omics modules distributed across six tissues, with none identified in the kidney (Supplementary Data S3, Table S4). The remaining 286 input metabolites (76.7%) were not assigned to a metabolite‐containing cross‐omics module. Topological analysis showed that 13 of these 49 modules contained at least one metabolite that served as an articulation point [45], whose removal increased the number of connected components, indicating a metabolite‐bridging role, whereas only six contained exclusively peripheral metabolites, each with a degree of one (Supplementary Data S3, Tables S5 and S6 and Supplementary Methods). Among these 49 modules, 21 simultaneously contained transcriptomic, proteomic, and metabolomic features, with 3 to 65 molecular features per module (median = 22; mean = 27.33; Figure 5a). The confidence scores across these modules had a median and mean of 0.76 (Figure 5a and Supplementary Data S3, Table S7).

Among these modules, two thymus‐associated modules recapitulated the previously reported decline in translation‐associated programs during aging [3, 46]. One module directly captured downregulation of “Ribosome biogenesis and RNA processing” (Supplementary Figure S13a). Beyond this known aging‐associated translation decline, MAPA further linked these changes to coordinated upstream metabolic and signaling alterations through a second module annotated as “Regulation of mTOR signaling and methionine metabolism” (Supplementary Figure S13b). This module integrated molecular components acting upstream of mTORC1 signaling [47, 48, 49, 50, 51, 52], suggesting coordinated suppression of mTOR‐associated metabolic signaling during thymic aging.

MAPA further identified coherent proteoglycan‐associated functional programs from both single‐omics and multi‐omics perspectives (Figure 5b,c). In contrast to fragmented pathway‐level signals from individual omics layers, the multi‐omics module revealed a unified glycosaminoglycan and proteoglycan metabolic program composed of metabolites, age‐upregulated genes and pathways involved in glycosaminoglycan biosynthesis and modification. For example, the metabolite cytidine‐5’‐monophosphate (CMP) was integrated into the module through its reaction with a sialyltransferase encoded by ST3GAL (Figure 5c). This integration is supported by the role of CMP in forming the activated sugar donor [53]. These integrated signals collectively suggest increased glycosaminoglycan and proteoglycan metabolic activity in the aged aortic arch [54].

MAPA also detected a spleen functional module centered on the regulation of hemostasis and inflammation from both single‐omics and multi‐omics analyses (Figures 5d,e). In the single‐omics results, the transcriptomic module grouped glutamate‐cysteine ligase catalytic subunit (GCLC) into lipid catabolism, whereas the proteomic module mainly captured hemostasis‐ and inflammation‐related pathways (Figure 5d). In contrast, the multi‐omics module provided a more integrated view by linking a hemostasis/inflammation branch with a glutathione‐related branch (Figure 5e). In particular, MAPA grouped GCLC, glutamate‐cysteine ligase regulatory subunit (GCLM), sulfiredoxin‐1 (SRXN1), and peroxiredoxin‐2 (PRDX2) together with the metabolite γ‐glutamylcysteine, highlighting a coordinated antioxidant program that could not be captured by single‐omics analysis alone. This organization is biologically plausible, as GCLC, GCLM, and SRXN1 are targets of nuclear factor erythroid 2‐related factor 2 (NRF2), a key regulator of oxidative stress defense [55]. These results suggest that MAPA identified a coordinated NRF2‐associated antioxidant signal connected to the hemostatic and inflammatory program in the aged spleen.

Collectively, these multi‐omics functional modules show that MAPA can integrate gene, protein, and metabolite changes together with enriched pathways through knowledge‐driven connections, and provide coherent biological interpretations across different omics layers.

## 3 Discussion

In this study, we developed MAPA, a computational framework designed to improve the biological interpretation of multi‐omics data at the functional module level. By integrating established computational approaches, MAPA provides two major advances at the framework level. First, it integrates molecular interactions and pathway‐level functional context into a semantic‐biological network, allowing functional relatedness among genes, proteins, metabolites, and pathways to be quantified within a unified analytical space. Second, MAPA provides structured functional interpretations for identified modules using a retrieval‐augmented LLM strategy. These advances support MAPA as a general framework for organizing heterogeneous multi‐omics molecules into coherent functional modules and generating comprehensive biological interpretations.

To enable pathway‐level functional context to be incorporated into multi‐omics module discovery, MAPA uses biotext embedding similarity to quantify functional similarity between pathways. Conventional similarity metrics [37], such as the Jaccard index or overlap coefficient, rely on shared membership, limiting their sensitivity when functionally related pathways share few molecular components. In contrast, biotext embedding similarity captures semantic relationships encoded in pathway names and descriptions, enabling the detection of functional relatedness that is not reflected by component overlap alone. This property is particularly useful for cross‐database comparisons, where equivalent or closely related biological processes may be represented by distinct, non‐overlapping components. The higher discriminative performance of biotext embedding similarity (Cliff's δ = 0.96) supports its suitability as a database‐agnostic similarity measure across heterogeneous pathway resources (Figure 2b). This semantic foundation enables the downstream MAPA network to meaningfully connect pathways, genes, proteins, and metabolites across omics layers.

The benchmarking results further demonstrate the improved performance of MAPA in functional module identification. Under optimized settings, MAPA achieved higher module reconstruction accuracy on the curated pathway dataset (ARI = 0.95) than existing tools, including aPEAR [42], PAVER [27], and enrichplot [41] (ARI = 0.49‐0.67; Figure 3b). On the independently constructed informative GO term benchmark, MAPA remained the best‐performing method under both its default and optimized settings, with ARI increasing from 0.17 to 0.57 after parameter optimization. Parameter optimization also improved the performance of PAVER (0.15 to 0.49), aPEAR (0.02 to 0.13), and enrichplot (−0.001 to 0.11), indicating that clustering performance across methods can be strongly influenced by dataset‐specific parameter choices. Together, the two benchmarks suggest that a single default clustering configuration may not be uniformly optimal across datasets and that parameter selection should account for the underlying similarity structure and desired module granularity. To facilitate such dataset‐specific selection in practical applications, where predefined ground truth are generally unavailable, MAPA provides internal clustering‐quality metrics, such as the silhouette score, Calinski‐Harabasz index, and Davies‐Bouldin index, to support comparison among candidate parameter settings. For module interpretation, MAPA‐generated annotations outperformed those from alternative tools, and can capture biological context at a level approaching human expert interpretation while providing a more standardized and reproducible annotation workflow.

The case studies demonstrate the practical utility of MAPA in real biological contexts. In the single‐omics analysis, MAPA organized enrichment results from 78 tissues into interpretable functional modules, enabling the discovery of cross‐tissue aging patterns. Biologically coherent signatures, such as the consistent downregulation of FOXO signaling across peripheral tissues and tissue‐specific directionality of microRNA dysregulation, emerged as hypothesis‐generating findings that would not be readily apparent from conventional enrichment lists (Supplementary Figure S12a). In the multi‐omics case study, MAPA integrated transcriptomic, proteomic, and metabolomic signals into unified functional modules, revealing cross‐layer biological associations. For example, MAPA grouped GCLC‐related transcriptomic features, hemostasis‐associated proteomic signals, and an antioxidant metabolite into a single multi‐omics functional module, thereby capturing coherent functional themes that were not identified by single‐omics analyses alone (Figures 5d,e). These examples illustrate that multi‐omics analysis can provide qualitatively distinct and more comprehensive biological interpretations than single‐omics analysis.

The current framework also has several limitations. First, as a knowledge‐driven framework, MAPA relies on curated biological relationships; therefore, novel molecular relationships that are absent from existing knowledge bases cannot be directly discovered. This represents an inherent trade‐off in MAPA's design, which prioritizes interpretability, reduced dependence on large sample sizes, and accessibility without complex model training. The discovery of entirely new molecular patterns may be better addressed by complementary data‐driven approaches [4].

Second, rigorous benchmarking of module discovery and interpretation remains challenging because community‐accepted ground‐truth datasets for cross‐omics functional modules are not yet available. Although we constructed curated pathway benchmarks and used expert annotations, random controls, and real aging datasets to evaluate MAPA, these evaluations cannot fully replace experimentally validated functional module standards.

Third, MAPA depends on the quality of upstream omics analysis and molecular annotation. Differential feature selection, pathway enrichment, metabolite identification, identifier mapping, and database coverage can all influence the resulting modules. This limitation is particularly relevant for metabolomics, where incomplete structural annotation and limited pathway coverage may reduce the connectivity of metabolite features within the semantic‐biological network.

Finally, the LLM‐based annotation pipeline currently depends on external language models. Future development should therefore support local open‐source language models, such as Llama [56], Qwen [57], and DeepSeek [58], to reduce dependence on commercial APIs. Additional computational improvements, including embedding caching for large‐scale analyses and extensions to longitudinal data, could further improve scalability and expand MAPA's applicability to dynamic biological settings such as disease progression and treatment response.

In summary, MAPA provides a principled and practical framework for functional module discovery and interpretation in multi‐omics data. By combining semantically grounded similarity encoding with LLM‐powered annotation, MAPA addresses key gaps in existing approaches while remaining extensible to emerging biological and computational challenges.

## 4 Methods

### 4.1 Input Preparation

MAPA supports both single‐omics and multi‐omics inputs. For each omics layer, users may provide either a set of differential molecules for over‐representation analysis (ORA) [28] or a ranked list of molecules for gene set enrichment analysis (GSEA) [29]. Accepted identifiers include standard gene/protein identifiers (Ensembl, Entrez, UniProtKB, gene symbols) and metabolite identifiers (HMDB, KEGG). For transcriptomic and proteomic data, ORA and GSEA are performed using the clusterProfiler package [18] with Gene Ontology (GO) [34, 35], KEGG [32], and Reactome [33] as supported pathway databases. For metabolomics data, ORA is performed using the metpath package from the TidyMass project [59], using KEGG metabolic pathways and SMPDB (Small Molecule Pathway Database) [60] as default reference databases. In multi‐omics mode, differential molecules from all omics layers and enriched pathways from transcriptomic and proteomic data are used as input to the MAPA core workflow. In single‐omics mode, only enriched pathways are used.

### 4.2 Single‐Omics Pathway Encoding

For single‐omics input, MAPA represents enriched pathways using a text embedding strategy. Pathways passing user‐defined significance and size filters are first retained for downstream biotext embedding.

*Pathway text retrieval*. For each retained pathway, descriptive text is retrieved from the corresponding source database, including term names and definitions for GO, pathway titles and summaries for KEGG, and pathway names and overviews for Reactome. These elements are concatenated to generate a unified text representation of the pathway's functional context.

*Biological text embedding and pathway network construction*. The constructed pathway text is converted into a dense vector representation using a user‐specified embedding model accessed through API providers supported by MAPA, including OpenAI, Gemini, and SiliconFlow. Pairwise cosine similarities between pathway embeddings are then calculated to generate a pathway similarity matrix, which captures functional relatedness beyond direct gene overlap [61]. The pathway similarity matrix is further transformed into an undirected weighted network.

### 4.3 Multi‐Omics Network Construction and Encoding

For multi‐omics input, MAPA constructs a knowledge‐guided multi‐layer network that integrates transcriptomic, proteomic, and metabolomic features with enriched pathways, and encodes the functional relationships among molecules and pathways using diffusion profiles derived from random walk with restart (RWR).

*Biological relationship retrieval*. Curated biological relationships are retrieved from public knowledge bases to define connectivity across molecules and pathways. These relationships include transcription factor (TF)‐target regulatory interactions from DoRothEA [30], protein‐protein interactions from STRING [31], enzyme‐metabolite interactions and metabolite‐metabolite co‐reactions derived from KEGG and Reactome. Molecule‐pathway annotation associations are extracted from the enrichment results and used to connect molecules with enriched pathways.

*Network Construction*. The retrieved relationships are organized into a multiplex‐heterogeneous network composed of two heterogeneous networks (a molecular multiplex network and a monoplex pathway network) linked by the bipartite molecule‐pathway relationships. Construction of the network and all subsequent RWR computations were performed using the RandomWalkRestartMH R package [14]. These networks and relationships are defined as follows.

*Molecular multiplex graph*. The molecular multiplex graph consists of four layers defined over a common node set $V_{mol}=V_{t}\cup V_{prot}\cup V_{met}$, where |*V_mol_* | = *n*_1_ . Here, *V_t_*, *V_prot_*, and *V_met_* denote the sets of transcript, protein, and metabolite nodes, respectively. Each layer α  =  1,  2,  3,  4 encodes a distinct molecular relationship type: transcription factor‐target regulation, protein‐protein interaction, enzyme‐metabolite reaction association, and metabolite co‐reaction. Each layer is represented by a binary adjacency matrix $A_{mol}^{\left[\alpha \right]}={\left(A_{mol}^{\left[\alpha \right]}\;\left(i,j\right)\right)}_{i,j=1,\text{…},n_{1}}$, where an entry $A_{mol}^{\left[\alpha \right]}\left(i,j\right)=1$ indicates the presence of the corresponding relationship between two nodes, and 0 indicates its absence. The molecular multiplex graph is defined as *G_M_* =  (*V_mol_*,*E_mol_*), with

$$\begin{array}{ccc} V_{mol} & = & \left\{v_{i}^{\alpha }|i=1,\cdots ,n_{1},\alpha =1,2,3,4\right\},\cdot E_{mol}=\{\left(v_{i}^{\alpha },v_{j}^{\alpha }\right)|i,\\ j & = & 1,\cdots ,n_{1},\alpha =1,2,3,4,A_{mol}^{\left[\alpha \right]}\left(i,j\right)\neq 0\}\cup \{\left(v_{i}^{\alpha },v_{i}^{\beta }\right)|i=1,\\ & & \cdots ,n_{1},\alpha \neq \beta \}, \end{array}$$

where $v_{i}^{\alpha }$ denotes the node i in layer α.

*Pathway monoplex graph*. The pathway monoplex graph is defined over a set of enriched pathway nodes *V_path_*, where |*V_path_* | = *n*_2_ . Let *e_i_* denote the biotext embedding of pathway *i*. Pathway similarity is quantified by cosine similarity between the embeddings, and encoded in a weighted adjacency matrix $A_{path}\in R^{n_{2}\times n_{2}}$, defined as $A_{path}\left(i,j\right)=cos\left(e_{i},e_{j}\right)$ for i≠j and $A_{path}\left(i,i\right)=1$. The pathway monoplex graph is defined as *G_P_* =  (*V_path_*,*E_path_*), with

$$V_{path}=\left\{v_{i}|\;i=1,\text{…},n_{2}\right\},$$

$$E_{path}=\left\{\left(v_{i},v_{j}\right)|1\leq i<j\leq n_{2},A_{path}\left(i,j\right)\neq 0\right\}.$$

*Bipartite Pathway‐molecule Associations*. The Molecular Multiplex Graph and the Pathway Monoplex Graph Are Linked by Their Bipartite Association *E_B_*, Where

$$E_{B}=\left\{\left(v_{i},v_{j}\right)\;|\;1\leq i\leq n_{1},1\leq j\leq n_{2}\;\right\}\;.$$

Here $v_{i}\in V_{mol}$ denotes molecular node *i*, $v_{j}\in V_{path}$ denotes pathway node *j*. The corresponding association weight is defined as $w_{ij}=a_{ij}\;log\left(\frac{N_{bg}+1}{N_{j}+1}\right)$, where *a_ij_* =  1 if molecule *i* is annotated to pathway *j*, and 0 otherwise, *N_bg_* denotes the total number of background molecules, and *N_j_* denotes the number of background molecules annotated to pathway *j*.

*Multiplex‐heterogeneous network*. The complete multiplex‐heterogeneous network is obtained by integrating the molecular multiplex graph and the pathway monoplex graph through the bipartite pathway‐molecule associations. It is defined as *G_MP_* =  (*V_MP_*,*E_MP_*), with

$$V_{MP}=V_{mol}\cup V_{path},$$

$$E_{MP}=E_{mol}\cup E_{path}\cup \cup_{\alpha =1}^{4}E_{B}^{\left[\alpha \right]}.$$

where

$$E_{B}^{\left[\alpha \right]}=\left\{\left(v_{i}^{\alpha },v_{j}\right)\;|\;1\leq i\leq n_{1},1\leq j\leq n_{2},w_{ij}\rangle 0\right\}\;.$$

Here, $v_{i}^{\alpha }\in V_{mol}$ denotes a molecular node *i* in layer α, and $v_{j}\in V_{path}$ denotes pathway node *j*. Thus, each pathway node is connected to the corresponding layer‐specific copy of a molecular node whenever a bipartite association exists. The edge set ​*E_mol_* contains both within‐layer molecular edges and inter‐layer identity edges as defined above, *E_path_* ​ encodes pathway‐pathway similarity edges, and $E_{B}^{\left[\alpha \right]}$​ represents the bipartite associations between the molecular layer α and the pathway graph. The corresponding edge weights are given by the molecular adjacency matrices $A_{mol}^{\left[\alpha \right]}$​, the pathway similarity matrix $A_{path}$​, and the bipartite association weights $w_{ij}$, respectively. Then, a global adjacency matrix can be constructed by integrating $A_{mol}^{\left[\alpha \right]}$, $A_{path}$, and $w_{ij}$, thereby defining the global structural space of the multiplex‐heterogeneous network. This matrix was subsequently normalized to obtain the transition matrix used for random walk with restart.

*Diffusion profile generation by random walk with restart*. The resulting multiplex‐heterogeneous network *G_MP_* is then used to construct a global transition matrix *T*, which encodes the probability of the random walker moving between any two nodes in the network at each step.

The transition matrix is assembled in three components. First, for each molecular layer, an intra‐layer and inter‐layer adjacency block is constructed: within‐layer transition probabilities are scaled by (1 − δ), while inter‐layer (identity) transitions between copies of the same node across layers are scaled by δ/(*L* − 1), with *L* the number of layers (default 4). The parameter δ (default 0.5) governs the probability of switching between molecular layers, with a value of 0.5 assigning equal probability to intra‐ and inter‐layer transitions. Second, within‐layer pathway‐pathway transition probabilities are derived from the adjacency matrix $A_{path}$. Third, cross‐network transitions between molecular nodes and pathway nodes are weighted by the bipartite association weights $w_{ij}$, scaled by λ (default 0.5), which balances the probability of the walker staying within vs. jumping across networks. Each sub‐block of this global adjacency matrix is then column‐normalized to yield the final transition matrix *T*, ensuring that at each step the walker's probability distribution sums to one.

RWR is then performed iteratively. Starting from a seed node (either a molecule or an enriched pathway), the probability distribution over all nodes is updated at each step according to:

$$p_{t+1}=\left(1-r\right)Tp_{t}+rp_{0},$$

where *p_t_* is the probability vector at step *t*, *r* is the restart probability (default 0.7), and *p*_0_ is the seed vector initialised to 1 at the single seed node and 0 elsewhere. The layer weight vector τ defines the relative probability of restarting in each molecular layer's copy of the seed, and defaults to a uniform distribution (τ_α_ =  1/*L* for all layers) but can be adjusted to up‐ or down‐weight specific interaction types. Since the pathway network comprises a single layer, its corresponding restart weight is fixed at 1. At each step, the walker either traverses an edge according to *T*—moving within a molecular layer, jumping to the same node in another molecular layer, or crossing to the pathway network via a bipartite association—or restarts at the seed with probability *r*. This restart mechanism prevents the walker from becoming trapped in locally dense regions and anchors the exploration to the seed's neighborhood. Iteration continues until convergence, defined as ∥*p*_*t* + 1_ − *p_t_* ∥_2_ < ∈ (with ∈ = 10^−10^). When a molecular node appears across multiple layers, its layer‐specific scores are aggregated using the geometric mean, which penalizes nodes with high scores in only a subset of layers and reward those that are consistently proximal across multiple relationship types.

This procedure is repeated with each molecule and pathway node used as the seed in turn. The converged score vector for each seed constitutes its diffusion profile: a vector of length equal to the total number of nodes in *G_MP_*, where each entry quantifies the global network proximity between the seed and every other node. Biologically, the diffusion profile captures the functional context of a molecule or pathway as propagated through the integrated multi‐omics network: a molecule that is highly co‐regulated, participates in shared pathways, and is embedded in densely connected interaction subgraphs will exhibit high proximity to functionally related nodes. These diffusion profiles thus serve as high‐dimensional, network‐informed functional embeddings that simultaneously integrate transcriptomic, proteomic, and metabolomic relationships, and form the basis for subsequent pairwise similarity computation and functional module discovery.

### 4.4 Similarity‐Based Functional Module Discovery

After encoding, MAPA computes pairwise functional similarity between pathways or nodes and uses the resulting similarity structure for functional module discovery. In the single‐omics setting, pairwise pathway similarity is computed from pathway biotext embeddings, as described above. In the multi‐omics setting, pairwise similarity among molecules and pathways is computed from their diffusion profiles derived from the multi‐layer network. Specifically, cosine similarity is calculated between the diffusion profiles of all node pairs, generating a similarity matrix that quantifies their functional relatedness.

*Functional module discovery*. The similarity matrix is then used for functional module discovery. MAPA supports three major classes of clustering algorithms. First, network‐based community detection methods can be applied to a similarity network constructed from the similarity matrix, with the Louvain algorithm used by default (Supplementary Methods). Alternative algorithms implemented in the igraph R package [62], including Girvan‐Newman, Leiden, and Infomap, are also supported. Second, MAPA provides distance‐based hierarchical clustering methods that operate on a distance matrix transformed from similarity values as 1 − *similarity*. Third, MAPA supports binary cut clustering, a recursive divisive algorithm designed for similarity matrices [36]. Across methods, users can control module granularity through the similarity threshold, and clustering results can be evaluated using MAPA's quality assessment functions.

*Clustering quality assessment*. To facilitate the selection of an appropriate similarity threshold, MAPA provides a clustering quality assessment function that summarizes module structure using standard evaluation metrics. Reported statistics include the number of modules, the proportion of non‐singleton modules, the average silhouette score [63], the Calinski‐Harabasz Index [64], and the Davies‐Bouldin Index [65]. In addition, MAPA generates module size distribution and silhouette width plots to visually assess cluster balance, cohesion, and separation, enabling systematic comparison of parameter settings.

### 4.5 Functional Module Interpretation

After functional modules are identified, MAPA performs automated annotation using an LLM‐assisted pipeline that integrates pathway metadata, biomedical literature, and optional user‐provided context to generate structured module‐level interpretations. The overall workflow is shared between single‐omics and multi‐omics analyses, with the main difference being the module content carried through the annotation process (Supplementary Figure S1). In single‐omics analysis, each module contains enriched pathways from one omics type. In multi‐omics analysis, each module jointly contains pathways, genes/proteins, and metabolites, enabling cross‐omics interpretation. MAPA supports multiple LLM platforms for annotation, including OpenAI GPT models, Google Gemini models, and SiliconFlow models.

*Literature collection and relevance ranking*. For each module, MAPA collects pathway descriptions, including GO term names and definitions, KEGG pathway titles and descriptions, and Reactome pathway names and summaries (Supplementary Figure S1a). Module‐associated molecular components are then compiled according to the analysis mode. For single‐omics modules, these components consist of a single molecular type linked to the module, either genes/proteins or metabolites. For multi‐omics modules, genes/proteins and metabolites are combined with pathway information. The resulting text is used to construct module representations for downstream literature filtering.

Relevant literature is retrieved by combining keyword‐based PubMed queries, pathway‐linked references from curated databases, and user‐uploaded text materials (Supplementary Figure S1a). In single‐omics analysis, PubMed queries are built from pathway terms together with a single molecular type. In multi‐omics analysis, pathway terms, gene/protein information, and metabolite names are queried jointly.

Retrieved abstracts and module representations are embedded using a biotext embedding model, and cosine similarity is used to select the top 20 candidate papers by default. These candidates are further re‐ranked using an LLM with a structured prompt designed to assess biological relevance to the module (Supplementary Note S6). The top 5 papers by default are retained as supporting literature for downstream interpretation.

*Prompt engineering for functional module summarization*. The final summarization step uses a structured prompt to generate a functional summary for each module (Supplementary Figure S1b). For single‐omics modules, the prompt summarizes pathways together with one molecular type (Supplementary Note S6). For multi‐omics modules, the prompt explicitly incorporates pathways, genes/proteins, and metabolites (Supplementary Note S6). The LLM is instructed to produce a standardized JSON output containing: (i) a concise and biologically meaningful module name; (ii) a summary describing the core biological processes represented by the module; (iii) an optional phenotype‐specific interpretation; and (iv) a confidence score (0.00‐1.00) reflecting the coherence and relevance of the annotation. This score is a heuristic assessment of how strongly the module components and supporting literature converge on a single biological interpretation. When phenotype information is provided, it also considers the support and mechanistic plausibility of the module‐phenotype relationship. It is not a calibrated probability of annotation correctness. For multi‐omics modules, the summary is further expected to highlight cross‐omics consistency and functional links across pathways, genes, proteins, and metabolites. This design enables MAPA to perform rich, literature‐informed interpretation of both single‐omics and multi‐omics functional modules in a consistent format, offering greater interpretability than conventional single‐pathway‐based summaries.

### 4.6 Result Visualization and Reporting

MAPA provides an integrated output framework that combines multi‐level visualization with automated reporting to facilitate interpretation, comparison, and downstream analysis of functional module results.

*Multi‐level visualization of functional modules*. MAPA visualizes pathway enrichment and functional module results using standardized plots and networks. Enrichment results from ORA and GSEA can be displayed at the pathway, module, and functional module levels to facilitate comparison of statistical significance and functional relevance. Functional modules are represented as network graphs. In particular, multi‐omics functional modules can be visualized as integrated networks that simultaneously show curated knowledge‐based edges and RWR‐inferred functional associations, enabling the exploration of relationships among cross‐omics molecular features and pathways within each module. For integrative interpretation, MAPA supports combined visualization of functional relationships and molecular expression patterns, allowing users to link module structure with underlying molecular activity.

*Result report*. MAPA generates standardized analysis reports using automated templates. Reports summarize key analysis parameters, major enrichment results, functional module organization, and interpretation outputs when available. All intermediate and final results can also be exported in structured tabular formats, ensuring compatibility with downstream analyses and custom visualization workflows.

### 4.7 Methodological Evaluation of MAPA

To systematically assess the robustness, accuracy, and interpretability of MAPA, we performed a comprehensive methodological evaluation covering multiple components of the framework. Specifically, we evaluated the effectiveness of biotext embedding‐based pathway similarity, the performance of different clustering strategies for functional module discovery, the quality of functional interpretation, and whether MAPA confidence scores were sensitive to differences in module functional coherence. We further assessed robustness to an alternative embedding and language model and evaluated MAPA on an independently constructed informative GO term benchmark dataset, including parameter‐sensitivity analysis. Finally, the stability and reproducibility of MAPA results were evaluated across repeated runs. These evaluations were designed to ensure that MAPA produces biologically meaningful, robust, and reproducible functional modules. Detailed evaluation procedures are provided in Supplementary Methods.

### 4.8 Benchmarking MAPA With Existing Methods in the Single‐omics Setting

We compared *MAPA* with three representative established methods: enrichplot [41], PAVER [27], and aPEAR [42]. The comparison evaluated two key aspects: functional module discovery and interpretation quality. Input data requirements, clustering algorithms, and interpretation methods for these tools are detailed in Supplementary Table S2. Benchmarking was performed using both the curated pathway dataset comprising 44 pathways assigned to 12 predefined functional modules (Supplementary Methods and Supplementary Data S1, Table S1) and an independently constructed informative GO term dataset comprising 313 terms assigned to 57 modules (Supplementary Methods and Supplementary Data S2, Tables S1 and S2). The latter provided an external benchmark with module assignments defined systematically from the GO hierarchy. For methods requiring enrichment input, all pathways or GO terms were treated as enriched terms, and an artificial enrichment object was generated using the clusterProfiler package.

*Clustering quality comparison*. Both default and optimized configurations were evaluated for each method. The default MAPA configuration used Louvain community detection with a biotext embedding similarity threshold of 0.55. The default PAVER configuration used Ward's hierarchical clustering (ward.D2), minClusterSize = 20, and the default dynamicTreeCut settings. The default aPEAR configuration used Jaccard pathway similarity, Markov clustering, and minClusterSize = 2. The default enrichplot configuration used the Kamada‐Kawai layout (layout = “kk”), min_edge = 0.2, K‐means clustering, and $\sqrt{N}$ clusters, where *N* denotes the number of pathways or GO terms.

For optimized analyses, method‐specific parameter searches were performed separately for each benchmark dataset. For MAPA, we systematically evaluated graph‐based community detection, distance‐based clustering, and binary cut approaches across selected similarity thresholds, cluster numbers, and algorithm‐specific parameters. The best‐performing configuration on the curated pathway dataset was Louvain clustering with a biotext embedding similarity threshold of 0.55, which was subsequently adopted as the default MAPA configuration (Supplementary Methods). On the informative GO dataset, the optimized MAPA result was obtained using K‐means clustering with 77 centers. PAVER was optimized across hierarchical linkage methods and dynamic‐tree‐cut parameters; aPEAR across pathway‐similarity measures, clustering algorithms, and minimum cluster sizes; and enrichplot across network‐layout, edge‐threshold, and cluster‐number settings. Because the tools expose different clustering parameters, the candidate search spaces were method‐specific and restricted to the clustering‐relevant parameters described above. Parameters not included in the search were retained at their default values. Across methods, the same selection criterion was used, with the configuration achieving the highest adjusted Rand index (ARI) relative to the ground‐truth modules designated as the optimized result. Detailed parameter search spaces are provided in Supplementary Data S2, Table S5, while the default and selected optimized configurations for each method on both benchmark datasets, together with their corresponding ARIs, are summarized in Supplementary Data S2, Table S6. For visualization, the overlap between every identified module and every ground‐truth module was calculated using the Jaccard index, $J\left(A,B\right)=\frac{\left|A\cap B\right|}{\left|A\cup B\right|}\;$, with all identified modules retained in the heatmaps.

*Functional module interpretation comparison*. Interpretation performance was evaluated using the curated pathway dataset by applying each method's interpretation strategy to the ground truth clustering. enrichplot generates module labels based on word frequency and positional information in pathway names, aPEAR selects representative pathways using PageRank, and PAVER identifies the pathway whose embedding shows the highest cosine similarity to the module‐average embedding. MAPA generates functional module names and summaries using an LLM‐assisted interpretation framework. All interpretations were converted to text embeddings and compared against expert interpretations using cosine similarity.

### 4.9 Applying MAPA to Two Aging‐Related Multi‐omics Datasets

*Single‐omics analysis case study*. This dataset is from a previously published study [1] investigating aging and metformin treatment effects in male cynomolgus monkeys across different age groups (3‐5, 10–12, and 16–19 years). Detailed sample preparation and analytical conditions are described in the original study. Briefly, the multi‐omics data includes bulk RNA sequencing data from 79 tissues using the MGI DNBSEQ‐T7 platform, plasma proteomics data using Data Independent Acquisition (DIA) on Thermo Scientific Q Exactive HF mass spectrometer, and plasma metabolomics data using UPLC‐MS/MS on ACQUITY 2D UPLC system paired with Q Exactive mass spectrometer.

Age‐dependent molecular changes were characterized using fuzzy c‐means clustering (Mfuzz R package), revealing four distinct temporal patterns: continuous upregulation (Cluster U), continuous downregulation (Cluster D), initial upregulation followed by downregulation (Cluster UD), and initial downregulation followed by upregulation (Cluster DU). The reported Mfuzz‐derived gene‐expression trend results were available for only 78 of the 79 tissues. Accordingly, our transcriptomic analysis used the age‐dependent gene lists from Clusters U and D for these 78 tissues. The corresponding age‐dependent protein and metabolite lists from Clusters U and D were used for the plasma proteomic and metabolomic analyses.

MAPA clustered enriched pathways from each omics layer into tissue‐specific functional modules with default parameters. For transcriptomic data, to quantify the directional trend of each module with age, we calculated a Dysregulation Index defined as follows:

$$Dysregulation\;Index=\frac{N\left(cluster\;U\right)-N\left(Cluster\;D\right)}{N\left(cluster\;U\right)+N\left(Cluster\;D\right)},$$

where *N*(*cluster* *U*) is the number of upregulated genes and *N*(*cluster* *D*) is the number of downregulated genes within a given functional module. A higher index indicates that the module is predominantly upregulated during aging, whereas a lower index indicates downregulation.

Functionally conserved patterns across tissues were further identified by clustering tissue‐specific modules into higher‐level meta‐modules based on the semantic similarity of their functional interpretation (hierarchical clustering, similarity cutoff = 0.55). For each meta‐module, we generated a comprehensive functional interpretation by synthesizing the summaries and tissue origins of all its constituent modules using an LLM prompt (Supplementary Note S6). The resulting meta‐modules revealed shared functions exhibiting consistent age‐associated regulation across tissues. These patterns were visualized using a heatmap summarizing module‐level dysregulation trends.

We further extended this analysis to plasma proteomics and metabolomics data, demonstrating the general applicability of MAPA across omics layers.

*Multi‐omics analysis case study*. We applied MAPA to a publicly available aging‐related multi‐omics dataset generated from a previously published study of female rhesus macaques (*Macaca mulatta*) [3]. Detailed sample preparation and analytical conditions are described in the original study. Briefly, the multi‐omics data includes transcriptomic, proteomic, and metabolomic profiles from 30 solid tissues of 17 female rhesus macaques aged 3–27 years. The transcriptomic data were generated using RNA sequencing on the Illumina NovaSeq 6000 platform, proteomic data were generated using LC‐MS/MS in data‐dependent acquisition and data‐independent acquisition modes on a Q‐Exactive HF‐X mass spectrometer, and metabolomic data were generated using UHPLC‐MS/MS on an Orbitrap Q‐Exactive HF‐X mass spectrometer. Tissue‐specific age‐associated molecular changes were identified using the linear regression model of “molecular abundance ∼ age”. For our analysis, age‐upregulated (*p* value < 0.05 and age‐effect estimate > 0.008) and age‐downregulated (*p* value < 0.05 and age‐effect estimate < ‐0.008) genes, proteins, and metabolites were used together as input for the MAPA multi‐omics analysis.

To focus on tissues with pronounced age‐related molecular remodeling and independent histological support, we selected the multi‐omics datasets from seven tissues that were further validated by immunofluorescence staining in the original study, namely spleen, thymus, aortic arch, ovary, thyroid gland, stomach, and kidney. Age‐upregulated and age‐downregulated genes/proteins were used together with age‐dysregulated metabolites as input for the MAPA multi‐omics analysis. Metabolite‐containing cross‐omics modules (≥ 2 molecular omics types, including metabolomics and module size ≥ 3) were identified in all tissues except the kidney. Matched single‐omics analyses were additionally performed for the aortic arch, spleen, thymus, and thyroid gland to enable direct comparison between single‐omics and multi‐omics module organization and interpretation.

Database versions or resource identifiers and access dates used in the benchmarking and case study analyses, together with the fixed database snapshots and real‐time access modes supported by the current MAPA implementation, are summarized in Supplementary Data S4, Tables S1 and S2.

## 5 Code Availability

All software development, data processing, and statistical analyses were conducted using R version 4.5.0 and its associated packages (Supplementary Note S7). The complete source code for MAPA and MAPAShiny is freely available on GitHub at https://github.com/jaspershen‐lab/mapa and https://github.com/jaspershen‐lab/mapashiny, and has been archived in Zenodo (https://doi.org/10.5281/zenodo.17677509 and https://doi.org/10.5281/zenodo.17677547). All scripts used for data analysis and visualization in this study are available at https://github.com/jaspershen‐lab/mapa_manuscript and are archived in Zenodo (https://doi.org/10.5281/zenodo.17677544). Docker images for MAPA and MAPAShiny are provided via Docker Hub at https://hub.docker.com/r/jaspershenlab/mapa and https://hub.docker.com/r/jaspershenlab/mapashiny, enabling reproducible execution across platforms. Comprehensive tutorials and user documentation for both MAPA and MAPAShiny are available at https://www.shen‐lab.org/mapa‐tutorial/, with ongoing updates and maintenance information provided at https://www.shen‐lab.org/mapa‐website/. A fully hosted online instance of MAPAShiny is accessible at https://mapashiny.jaspershenlab.com.

## Author Contributions

XS conceived the project and provided overall supervision. YG, XS, and FZ collaboratively developed the methodology and software. MAPAShiny and its Docker implementation were developed by YG and YL. Documentation and tutorials of MAPA were prepared by YG and YS. Case study analyses were conducted by YG, CW, and XS, who also generated all figures. The manuscript was written by XS, CW, YG, and FZ, with all authors reviewing and contributing to the final version.

## Conflicts of Interest

The authors declare no conflicts of interest.

## Supporting information




**Supporting File 1**: advs77774‐sup‐0001‐SuppMat.docx.

**Supporting File 2**: advs77774‐sup‐0002‐SuppMat.docx.

**Supporting File 3**: advs77774‐sup‐0003‐SuppMat.xlsx.

**Supporting File 4**: advs77774‐sup‐0004‐SuppMat.xlsx.

**Supporting File 5**: advs77774‐sup‐0005‐SuppMat.xlsx.

## Data Availability

All the datasets analyzed in this study and used as demo data were obtained from the previously published studies [1, 3, 66]. All other source data supporting the findings are provided as Supplementary Data.
