# Supplementary material for: MAPA: A Semantic Network Framework for Functional Module Discovery and Interpretation in Multi‐Omics Data
Source: Adv Sci (Weinh). 2026 Sep 16:e77774. Online ahead of print. doi: 10.1002/advs.77774 (PMC13583226; doi:10.1002/advs.77774)
Supplement: Supplementary file 1 [file ADVS-9999-e77774-s002.docx]

**Supplementary Information for**

**MAPA: a semantic network framework for functional module discovery and interpretation in multi-omics data**

Yifei Ge^1,2,+^, Feifan Zhang^1,2,+^, Yijiang Liu^1,2,+^, Chao Jiang^3^, Peng Gao^4^, Nguan Soon Tan^1,5^, Sai Zhang^6^, Yuchen Shen^1,7^, Qianyi Zhou^8^, Xin Zhou^8^, Xiao Wang^9^, Fangqing Zhao^10^, Chuchu Wang^1,*^, and Xiaotao Shen^1,2,11,*^

1. Lee Kong Chian School of Medicine, Nanyang Technological University, Singapore, 639798, Singapore
2. School of Chemistry, Chemical Engineering and Biotechnology, Nanyang Technological University, Singapore, 639798, Singapore
3. Life Sciences Institute, Zhejiang University, Hangzhou, Zhejiang, 310058, China
4. Department of Environmental Health and Department of Molecular Metabolism, Harvard T.H. Chan School of Public Health, Boston, MA, 02115, USA
5. School of Biological Sciences, Nanyang Technological University, Singapore, 639798, Singapore
6. Department of Biomedical Informatics & Data Science, Yale University School of Medicine, Yale University, New Haven, CT, 06510, USA
7. Centre of Biomedical Systems and Informatics, International Campus, ZJU-UoE Institute, Zhejiang University School of Medicine, Zhejiang University, Haining, Zhejiang, 310058, China
8. Intelligent Medicine Institute, Fudan Microbiome Center, Fudan University Shanghai Medical College, Fudan University, Shanghai, 200433, China
9. State Key Laboratory of Crop Stress Adaptation and Improvement, State Key Laboratory of Cotton Bio-breeding and Integrated Utilization, Henan Joint International Laboratory for Crop Multi-Omics Research, School of Life Sciences, Henan University, Kaifeng, Henan, 450046, China
10. State Key Laboratory of Animal Biodiversity Conservation and Integrated Pest Management, Institute of Zoology, Chinese Academy of Sciences, Beijing, 100101, China
11. Singapore Phenome Center, Nanyang Technological University, Singapore, 639798, Singapore

^+^ These authors contributed equally: Yifei Ge, Feifan Zhang, and Yijiang Liu.

^*^ Corresponding authors: Chuchu Wang ([chuchu.wang@ntu.edu.sg](mailto:chuchu.wang@ntu.edu.sg)) and Xiaotao Shen ([xiaotao.shen@ntu.edu.sg](mailto:xiaotao.shen@ntu.edu.sg)).

## Table of Contents

## Supplementary Methods

## Supplementary Figure 1. Overview of the LLM-based functional module annotation pipeline in MAPA.

**Supplementary Figure 2.** Representative MAPA outputs generated from the package’s built-in example dataset (single-omics setting).

**Supplementary Figure 3.** Example MAPA structured report.

**Supplementary Figure 4.** Evaluation of clustering strategies and comparison between the curated and MAPA-derived module assignments.

**Supplementary Figure 5.** Pairwise correlation among different component-based similarity metrics.

**Supplementary Figure 6.** Discriminative power of similarity metrics specifically for GO terms.

**Supplementary Figure 7.** Reproducibility for MAPA’s performance across independent runs on curated data.

**Supplementary Figure 8.** Robustness of MAPA to alternative embedding and language models.

**Supplementary Figure 9.** Benchmarking MAPA against other pathway redundancy analysis tools on the curated pathway dataset.

**Supplementary Figure 10.** Benchmarking and clustering sensitivity analysis using the informative GO term dataset.

**Supplementary Figure 11.** MAPA identifies functional modules from age-related proteomics and metabolomics data.

**Supplementary Figure 12.** Shared age-related functional modules across diverse tissues identified by MAPA.

**Supplementary Figure 13.** MAPA identifies two thymus multi-omics modules associated with age-related translational decline.

**Supplementary Table 1.** Comparison of MAPA with existing multi-omics module discovery tools.

**Supplementary Table 2.** Comparison of MAPA with existing pathway clustering tools.

**Supplementary Note 1.** Key limitations of existing pathway similarity metrics.

## Supplementary Note 2. Existing pathway similarity metrics.

**Supplementary Note 3.** Biological connections among GO:0098985, GO:0098978, and hsa04724.

**Supplementary Note 4.** Instruction for human experts to generate the module functional annotation.

**Supplementary Note 5.** Explanation for differences between ground truth and clustering result generated by louvain algorithm (similarity cutoff: 0.55).

**Supplementary Note 6.** Prompt engineering.

**Supplementary Note 7.** R version and R packages used for data analysis in this study.

## Supplementary Methods

### Construction of the curated pathway dataset

To provide a ground truth for evaluating MAPA’s ability to cluster functionally related pathways, we constructed a curated pathway dataset. A single curator first selected functionally distinct GO terms from AmiGO2[^1^](https://www.zotero.org/google-docs/?pl1vLA) as seed terms. For each seed, the corresponding expected module was expanded using predefined database relationships, including immediate “is a” or “part of” parent and child GO terms and cross-referenced KEGG or Reactome pathways. When database cross-references were unavailable, KEGG or Reactome pathways with identical or synonymous names were manually matched to the corresponding GO seed module. No biotext embedding similarities, pairwise similarity matrices, or MAPA clustering results were inspected or used during seed selection, pathway mapping, or module assignment. The final dataset contained 12 expected modules, including 8 multi-pathway modules (3-7 pathways each) and 4 singletons, comprising 44 pathways in total (22 GO terms, 14 KEGG pathways, and 8 Reactome pathways) (Supplementary Fig. 4c and Supplementary Data 1, Table S1). This dataset served as the ground truth for clustering evaluation.

### Construction of the informative GO term dataset

To construct a larger and systematically defined benchmark dataset, we generated an informative GO term dataset from the GO Biological Process (BP) ontology using the GO basic ontology released on 19 June 2026 and the human UniProt-GOA annotation file generated on 18 June 2026. A directed GO BP graph was constructed using non-obsolete terms and “is_a” and “part_of” relationships. Human UniProt-GOA annotations were mapped to GO terms and propagated to ancestor terms through these relationships.

To define a biologically informative level of the GO hierarchy, we adopted the annotation-based informative-term criterion[^2^](https://www.zotero.org/google-docs/?z0YKrR). Very broad GO terms tend to represent general functions with limited specificity, whereas highly specific terms may contain too few annotated proteins for reliable analysis. Accordingly, an informative GO term was defined as a term associated with at least 20 propagated UniProt accessions while none of its direct child terms reached this threshold. This criterion balances functional specificity with annotation support and provides an annotation-defined resolution for module construction, avoiding the need to manually select a particular GO hierarchy level. Reference modules were defined directly from GO parent-child relationships. A GO term with at least four eligible informative GO terms as direct children was used to define a reference module. The parent term’s name and definition were used as the reference functional interpretation for that module. Informative terms linked to more than one parent term were excluded to ensure non-overlapping assignments. The final dataset contained 313 GO terms assigned to 57 ontology-defined modules, with 4-16 terms per module (median, 5) (Supplementary Fig. 10a).

### Evaluation of the biotext embedding similarity

To evaluate the effectiveness of the biotext embedding similarity implemented in MAPA, we benchmarked it against representative pathway similarity methods commonly used in pathway analysis. These methods were selected to cover distinct conceptual paradigms for defining pathway similarity (Supplementary Note 2). First, we compared biotext embedding with component-based similarity approaches, which quantify similarity based on overlap in annotated molecular components (e.g., genes, proteins, or metabolites). These methods capture direct content overlap but are sensitive to pathway size and annotation density. We excluded component-based methods with highly redundant correlation profiles and retained the Jaccard index and overlap coefficient as representative approaches (Supplementary Fig. 5). Second, we evaluated semantic similarity methods that leverage hierarchical relationships within controlled vocabularies. Such methods are widely used for GO terms and quantify similarity based on shared ontology structure. To ensure a fair and comprehensive comparison, all similarity methods were applied to the same benchmark dataset. Because semantic similarity methods are only applicable to GO terms, we adopted a two-stage evaluation strategy. In the first stage, the biotext embedding method and component-based methods were evaluated on the full dataset. In the second stage, the biotext embedding method was compared with both component-based and semantic similarity methods on GO terms only. This design enabled direct comparison across general pathway collections as well as GO-specific contexts.

### Evaluation of functional module interpretability using human expert annotations

To assess the interpretability of MAPA-generated functional module annotations, we compared them with annotations independently generated by five domain experts with relevant backgrounds. The five experts had complementary areas of expertise: Expert #1 (exposomics, microbiome science, and microbial evolution); Expert #2 (molecular biology, structural biology, and neuroscience); Expert #3 (environmental health, exposomics, and molecular metabolism); Expert #4 (microbiome medicine, inflammation, and metabolic disorders); and Expert #5 (human microbiome research, biostatistics, and machine learning). The experts were recruited by the corresponding author, were not authors of this study, and were not involved in MAPA development, construction of the curated benchmark, generation of MAPA annotations, or analysis of the benchmarking results. Their identities were known only to the corresponding author. The authors responsible for compiling and analyzing the annotations received only anonymized expert identifiers and general descriptions of their expertise.

Each expert received the anonymized module identifier together with the names and descriptions of the pathways and GO terms included in each module (Supplementary Data 1, Table S1), as well as the standardized instructions provided in Supplementary Note 4. They were not provided with the predefined module labels, MAPA-generated annotations or confidence scores, annotations from the other experts, or benchmarking results. All experts completed the annotation task independently. They were permitted to consult literature and biological databases but were instructed not to use large language models or other AI-based tools.

For quantitative comparison, the module names and functional summaries generated by MAPA and the experts were converted into text embeddings. Inter-expert semantic concordance was assessed by calculating pairwise cosine similarity among the five expert annotations for each module. When summarizing inter-expert semantic concordance, comparisons involving the annotation reported as “Not Available” by Expert #1 were excluded from the calculation of the median and IQR. Principal component analysis (PCA) was additionally applied to the annotation embeddings for each module to visualize the semantic relationships between MAPA-generated and expert annotations. Pairwise similarities were pooled across modules and summarized using the median and interquartile range (IQR).

### Evaluation of reproducibility and robustness

Reproducibility of the MAPA workflow was assessed across 15 independent runs on the curated pathway dataset. Clustering consistency was quantified using pairwise adjusted rand index (ARI)[^3^](https://www.zotero.org/google-docs/?bPafCA). To evaluate annotation stability, LLM-generated functional summaries were converted into text embeddings, and pairwise cosine similarities were computed for annotations of the same functional module across runs (to measure consistency), as well as between different modules (to measure specificity).

Robustness to model choice was further evaluated by repeating the curated benchmark using “Qwen3-Embedding-8B” for pathway embedding and “Qwen3.6-35B-A3B” for module interpretation, accessed through SiliconFlow. Pathway embeddings were regenerated using the alternative embedding model, and pairwise cosine similarities were compared with those obtained using the default embedding model. The resulting similarity matrix was clustered using the MAPA default configuration (Louvain, similarity cutoff = 0.55), and agreement with the curated ground-truth assignments was quantified using ARI. Qwen-generated module interpretations were additionally compared with expert annotations using text-embedding cosine similarity. The confidence score comparison between real and size-matched random modules was also repeated using the Qwen language model with the same analysis settings.

**Evaluation of confidence score specificity**

A negative control analysis was conducted using size-matched randomized pathway modules. For each curated module, a random module was generated by sampling pathways from outside the original module (Supplementary Data 1, Table S5). Real and random modules were processed using the same pipeline, and intra-module similarity and LLM-based module confidence scores were compared. The confidence-score comparison was additionally repeated using the alternative Qwen-based language model to assess robustness to model choice.

### Evaluation of clustering methods

We benchmarked multiple clustering strategies on the curated pathway dataset with known ground truth module assignments to evaluate their ability to identify functional modules (Supplementary Fig. 4). Three classes of clustering approaches were evaluated: graph-based community detection methods, distance-based clustering algorithms, and binary cut. For each method, relevant parameters were systematically tuned, and the configuration with the highest ARI was retained to enable fair comparison across the target range of 2 to 43 clusters (Supplementary Fig. 4a).

*Graph-based methods* For graph-based clustering, we constructed similarity networks by applying various cutoff thresholds (ranging from 0.2 to 0.9 in increments of 0.01) to filter edges based on similarity scores. We evaluated eleven community detection algorithms implemented in the igraph R package[^4^](https://www.zotero.org/google-docs/?cqNSpu): fast greedy, Louvain, walktrap, InfoMap, label propagation, leading eigenvector, Leiden, edge betweenness, optimal, spinglass, and fluid communities. Most algorithms operated directly on similarity weights, while the edge betweenness algorithm required distance measures and used converted weights calculated as distance = 1 – similarity. These algorithms automatically determine the optimal number of clusters based on modularity maximization rather than allowing direct specification of cluster numbers.

*Distance-based methods* We applied multiple distance-based clustering approaches to the pathway similarity matrix. For hierarchical clustering, we converted the similarity matrix to a distance matrix using distance = 1 – similarity and systematically tested eight linkage methods (ward.D, ward.D2, single, complete, average, mcquitty, median, centroid) across all possible cluster numbers from 2 to 43 using the hclust function with cutree to obtain desired cluster counts. K-means clustering was performed on similarity matrices, directly specifying cluster centers from 2 to 43 with 50 random starts (nstart = 50) for robust optimization. For methods that do not allow direct cluster number specification, including HDBSCAN (with minimum points parameters from 2 to 43), Affinity Propagation (with preference values from 0.2 to 0.9), Mean-Shift clustering (with bandwidth parameters from 0.05 to 1.0), and Gaussian Mixture Models (with component numbers from 2 to 43), we systematically varied their respective parameters and retained only the configuration with the highest ARI score.

*Binary cut* This was performed using the simplifyEnrichment R package[^5^](https://www.zotero.org/google-docs/?NqU0aG) with systematic evaluation of cutoff values ranging from 0.5 to 0.95 in increments of 0.01. Since binary cut automatically determines the number of clusters based on the similarity structure rather than allowing direct specification, we applied the *binary_cut* function with try_all_partition_fun = TRUE across all cutoffs and calculated ARI scores against ground truth labels.

Clustering agreement with ground truth was quantified using the ARI, as implemented in the mclust R package[^3^](https://www.zotero.org/google-docs/?wwbDZG). Statistical significance of performance differences between representative graph-based (Louvain) and distance-based (hierarchical clustering with ward.D2 linkage) approaches was assessed using paired permutation tests on ARI scores (9,999 permutations, α = 0.05) (Supplementary Fig. 4b).

The clustering sensitivity analysis was additionally repeated on the independently constructed informative GO term benchmark dataset (Supplementary Data 2, Tables S1-S2). The same classes of clustering strategies were evaluated. MAPA’s default configuration (Louvain clustering, similarity cutoff = 0.55) was evaluated without retuning, and optimized performance was determined by ARI-guided parameter search (Supplementary Fig. 10c-d and Supplementary Data 2, Table S5).

### Quantification and topological assessment of metabolite-containing cross-omics modules

For the seven-tissue multi-omics analysis, modules with molecular features from at least two molecular omics types were retained (module size ≥ 3). Omics types were determined from transcriptomic, proteomic, and metabolomic features, excluding pathway nodes. A metabolite-containing cross-omics module contained at least one metabolite together with at least one transcript or protein, whereas a three-omics module contained all three molecular omics types. Input metabolites were matched to module assignments by metabolite identifier and classified as assigned if they occurred in at least one metabolite-containing cross-omics module, or unassigned otherwise.

Topological analysis was performed on the full unweighted, undirected network of each metabolite-containing cross-omics module, including all molecular and pathway nodes and all retained edges. Metabolite degree and articulation-point status were calculated using the degree() and articulation_points() functions in the igraph R package. Modules containing at least one metabolite articulation point were classified as metabolite-bridged; modules without a metabolite articulation point but with at least one metabolite of degree ≥ 2 were classified as topologically integrated; and modules in which all metabolites had degree ≤ 1 were classified as peripheral.

### General statistics

All data processing, statistical analyses, and visualizations were conducted using R version 4.5.0 (<https://www.r-project.org/>) within the RStudio environment. A comprehensive list of R packages utilized in this study is provided in the Supplementary Note 7. Statistical significance was assessed using the Wilcoxon rank-sum test implemented via the ggsignif package and wilcox.test function[^6^](https://www.zotero.org/google-docs/?xFahaM). Multiple hypothesis testing was corrected using the False Discovery Rate (FDR) method (*p.adjust* function). Effect sizes were calculated to quantify the magnitude of the difference between groups. Specifically, we used the non-parametric statistic Cliff’s delta (δ), which ranges from -1 to 1, where 0 indicates stochastic equality and ±1 indicates complete dominance by one group[^7^](https://www.zotero.org/google-docs/?2BxuSc). Calculations were performed with the cliff.delta function from the effsize R package[^8^](https://www.zotero.org/google-docs/?P8W0gO). Dimensionality reduction was performed using PCA via the prcomp function and Uniform Manifold Approximation and Projection (UMAP) implemented through the uwot package[^9^](https://www.zotero.org/google-docs/?SkCJzs). Figure icons were obtained from iconfont.cn and are used under the terms of the MIT License for non-commercial purposes (<https://pub.dev/packages/iconfont/license>).

## Supplementary Figure 1. Overview of the LLM-based functional module annotation pipeline in MAPA. (a) Relevant publications are retrieved from PubMed, pathway database references, and optional user-uploaded literature, then prioritized by text-embedding similarity and LLM-based reranking. (b) The selected publications, pathway information, and optional experimental or phenotype context are integrated into a structured prompt, enabling the LLM to generate a functional module name, biological summary, phenotype-related interpretation, and confidence score.

## Supplementary Figure 2. Representative MAPA outputs generated from the package’s built-in example dataset (single-omics setting). (a) Module detail view combining the intra-module network, pathway enrichment summary, and a keyword cloud. (b) Similarity network of functional modules (size ≥3) illustrating relationships among modules. (c) Left, a multipartite graph linking molecules, pathways, and functional modules; right, an expression heatmap integrated with the relationship network and keyword clouds, jointly representing functional themes and expression patterns.

## Supplementary Figure 3. Example MAPA structured report. The report compiles: (1) package version and timestamps; (2) input parameters; (3) object information summarizing methods, databases queried, and processing steps; (4) bar plot of the top 10 enriched pathways or representative pathways from functional modules; (5) module network summarizing relationships among functional modules and enriched pathways; (6) an interpretation table listing module ID, functional name, summary, module-phenotype analysis, and confidence score generated by the large language model.

## Supplementary Figure 4. Evaluation of clustering strategies and comparison between the curated and MAPA-derived module assignments. (a) Heatmap showing the adjusted Rand index (ARI) achieved by different clustering algorithms across the resulting numbers of clusters. (b) Permutation distribution of the difference in ARI between Louvain clustering and hierarchical clustering with ward.D2 linkage. The red vertical line indicates the observed ARI difference. (c) Network representation of the 12 manually curated expected modules in the benchmark dataset. (d) MAPA’s module assignments generated by the Louvain algorithm using a biotext embedding similarity cutoff of 0.55. Louvain generated 14 modules and achieved an ARI of 0.95 relative to the curated assignments. The difference in module number arose because hsa04210 (Apoptosis) and hsa00190 (Oxidative phosphorylation) each formed a singleton module.

## Supplementary Figure 5. Pairwise correlation among different component-based similarity metrics. The scatterplot matrix compares five component-based similarity scores. Diagonal plots show the score distribution for each method, distinguishing between pairs from the same module (red) and different modules (dark grey). Off-diagonal plots show the pairwise correlations between metrics. Pearson correlation coefficients (Corr) are shown in the upper triangle, with asterisks denoting significance (*** *p* values < 0.001). The Jaccard index, Cohen's Kappa, and Dice coefficient are highly correlated (r > 0.97).

## Supplementary Figure 6. Discriminative power of similarity metrics specifically for GO terms. (a) Pairwise correlation among similarity metrics. The scatterplot matrix compares biotext embedding, ontology-based similarity metrics specifically for GO terms (Wang, Resnik, Rel, Jiang, and Lin), and component-based similarity scores (Jaccard, overlap coefficient, Cohen's Kappa, and Dice coefficient). Diagonal plots show the score distribution for each method, distinguishing within-module pairs (red) and between-module pairs (dark grey). Off-diagonal panels show pairwise relationships; the upper triangle reports Pearson correlations (Corr) with asterisks for significance (*** *p*-values < 0.001). The similarity scores generated by Wang, Resnik, Rel, Jiang, and Lin methods are highly correlated (r > 0.97). The Jaccard index, Cohen's Kappa, and Dice coefficient are highly correlated (r > 0.98). (b) Comparison of similarity scores for within-module (red) versus between-module (dark gray) GO-term pairs (within-module, n = 19; between-module, n = 77, for each metric). Cliff’s delta (δ) quantifies separation. Center lines indicate medians, boxes indicate the IQR, and whiskers extend to the most extreme values within 1.5 × IQR. Statistical significance was assessed using Wilcoxon rank-sum tests. *** p-value < 0.001. Biotext embedding achieves perfect discrimination (δ = 1.00, p < 0.001), outperforming Jaccard (δ = 0.87) and overlap coefficient (δ = 0.91). Wang semantic similarity also attains δ = 1.00 for computable GO term pairs but cannot be evaluated across GO sub-ontologies, resulting in substantial missing values and highlighting a practical limitation for cross-ontology comparisons.

## Supplementary Figure 7. Reproducibility for MAPA’s performance across independent runs on curated data. The curated pathway dataset was processed through MAPA’s clustering and LLM interpretation pipeline for 15 independent runs. Consistent clustering was observed across runs (ARI = 1, resulting in 14 functional modules). LLM-generated functional summaries were obtained for the 8 functional modules containing more than one pathway. For each of these modules, the pairwise cosine similarity of functional annotations across the 15 runs (same_fm) was compared to the cosine similarity of annotations between different functional modules across all runs (diff_fm). For each functional module, n = 105 same_fm comparisons and n = 1,575 diff_fm comparisons were included. Center lines indicate medians, boxes indicate the IQR, and whiskers extend to the most extreme values within 1.5 × IQR. Statistical significance was assessed using Wilcoxon rank-sum tests. *** p-value < 0.001. High cosine similarity (> 0.91) was observed for annotations of the same functional module across different runs, indicating strong reproducibility. In contrast, annotations between different functional modules exhibited low similarity.

## Supplementary Figure 8. Robustness of MAPA to alternative embedding and language models. (a) Comparison of pairwise biotext embedding similarities generated using the default “text-embedding-3-small” model and “Qwen3-Embedding-8B” across all pathway pairs in the curated benchmark dataset (n = 946). Pearson correlation is shown. (b) Comparison of MAPA confidence scores for real and size-matched random modules generated using “Qwen3.6-35B-A3B” (n = 8 paired modules). Lines connect the corresponding real and random modules, and point size indicates module size. (c) Comparison of functional interpretation similarity to expert annotations between the default “GPT-4o-mini model” and “Qwen3.6-35B-A3B” for each non-singleton functional module (n = 5 per box). Each point represents the semantic similarity between a model-generated interpretation and one expert annotation. Orange crosses indicate comparisons involving an expert annotation reported as “Not Available”. Box center lines indicate medians, boxes indicate the IQR, and whiskers extend to the most extreme values within 1.5 × IQR. Statistical significance was assessed using paired Wilcoxon tests. * *p*-value < 0.05; NS., not significant.

## Supplementary Figure 9. Benchmarking MAPA against other pathway redundancy analysis tools on the curated pathway dataset. Comparison of functional module assignments generated by MAPA, PAVER, enrichplot, and aPEAR under optimized parameter settings. Clustering performance was quantified using the adjusted Rand index (ARI) against the ground-truth module assignments. (a) MAPA similarity network (ARI = 0.95). Nodes represent pathways and are colored by identified functional modules; edges represent biotext embedding similarities, with edge width proportional to similarity. Node size reflects −log10(FDR-adjusted p value). (b) PAVER multidimensional scaling (MDS) representation (ARI = 0.67). Each point represents an identified functional module and is labeled by the representative pathway with the highest cosine similarity to the module-average embedding. (c) enrichplot similarity network (ARI = 0.51). Nodes represent pathways, edges indicate pathway similarity, and colored regions denote identified modules, which are annotated using word-frequency-based summaries of pathway names. (d) aPEAR similarity network (ARI = 0.49). Nodes represent pathways, edges indicate Jaccard similarity, and ellipses denote identified modules labeled by representative pathways selected by aPEAR.

## Supplementary Figure 10. Benchmarking and clustering sensitivity analysis using the informative GO term dataset. (a) Distribution of module sizes in the informative GO term benchmark, expressed as the number of GO terms per module. (b) Comparison of within-module and between-module GO-term similarities calculated using biotext embedding similarity (left) and the Jaccard index (right). Each box represents pairwise GO-term comparisons (same module, n = 832; different modules, n = 47,996). Numbers indicate group medians. Box center lines indicate medians, boxes indicate the IQR, and whiskers extend to the most extreme values within 1.5 × IQR. Statistical significance was assessed using Wilcoxon rank-sum tests; **** p < 0.001*. (c) Sensitivity analysis of MAPA clustering performance across different clustering algorithms and parameter settings. Box plots on the right summarize the ARI distribution across parameter settings for each algorithm. (d) Comparison of default and optimized clustering performance of MAPA, PAVER, aPEAR, and enrichplot on the informative GO term benchmark. Arrows indicate the change from default to optimized performance.

## Supplementary Figure 11. MAPA identifies functional modules from age-related proteomics and metabolomics data. MAPA was applied to lists of plasma proteins and metabolites[^10^](https://www.zotero.org/google-docs/?VFaIRE) to construct functional module similarity networks. (a) The network for age-upregulated proteins. (b) The network derived from both age-upregulated and age-downregulated metabolites. The network was generated using MAPA to provide a biologically meaningful view of the functional relationships between pathways. Each node represents an enriched pathway, where node size is proportional to the statistical significance, and the edge width reflects the biotext similarity between pathways.

## Supplementary Figure 12. Shared age-related functional modules across diverse tissues identified by MAPA. (a) Heatmap of dysregulation index for selected functional modules (columns) across aging tissues (rows), calculated from transcriptomic data using MAPA. Red indicates upregulation in the old group; blue indicates downregulation. (b) A downregulated functional module annotated as “FOXO signaling and longevity regulation”. These changes are consistently observed across multiple tissues, including the liver (median lobe), intestine (ileum), bronchus (lower), and supraspinatus tendon.

## Supplementary Figure 13. MAPA identifies two thymus multi-omics modules associated with age-related translational decline. (a) A multi-omics module annotated as “Ribosome biogenesis and translation regulation”. (b) A multi-omics module annotated as “Regulation of mTOR signaling and methionine metabolism”. Proteomic features included in these modules were derived from proteins downregulated with aging in the thymus. Edge weights in the networks indicate biotext embedding similarity for pathway-pathway edges, diffusion profile-based similarity for inferred edges without direct knowledge-base support, and a fixed weight of 1 for knowledge-supported relationships.

## Supplementary Table 1. Comparison of MAPA with existing multi-omics module discovery tools.

|  | | MAPA | OmicsNet[^11^](https://www.zotero.org/google-docs/?HOxRYS) | netOmics[^12^](https://www.zotero.org/google-docs/?fQVv35) | OmicsAnalyst[^13^](https://www.zotero.org/google-docs/?uDCmnb) | MintTea[^14^](https://www.zotero.org/google-docs/?7eTsib) |
| --- | --- | --- | --- | --- | --- | --- |
| Input | | Molecule lists | Molecule lists | Abundance tables | Abundance tables | Abundance tables |
| Network construction strategy | Node | Molecules and pathways | Molecules only | Molecules and pathways | Molecules only | Molecules and pathways |
|  | Edge | Knowledge-driven | Knowledge-driven | Hybrid | Data-driven | Data-driven |
| Functional similarity quantification | | Yes;  Network-based functional profile similarity | No | Yes;  Network-based seed proximity | Yes;  Data-driven statistical relationships | Yes;  Data-driven cross-omics co-variation and phenotype association |
| Module definition | | Global functional-profile-based modules | Local topology-based modules | Seed-centered proximity-based modules | Correlation-network-based modules | Phenotype-associated co-variation modules |
| Module annotation strategy | | Yes;  LLM-assisted automated annotation | No;  Need manual annotation | No;  Need manual annotation | No;  Need manual annotation | No;  Need manual annotation |

## Supplementary Table 2. Comparison of MAPA with existing pathway clustering tools.

| **Tools** | **Input: accepts a simple list** | **Pathway similarity calculation** | | | **Clustering methods** | **Annotation methods** | |
| --- | --- | --- | --- | --- | --- | --- | --- |
|  |  | Component-based similarity | Semantic similarity | Embedding-based similarity |  | Module name | Module summary |
| enrichplot[^15^](https://www.zotero.org/google-docs/?N03WPl) | No;  Requires pre-generated enrichment results from clusterProfiler. | Yes | Yes | No | A selection of standard algorithms (e.g., k-means, PAM) that require the user to pre-specify the number of clusters. | Yes;  Selects top words based on word frequency within pathway names. | No |
| aPEAR[^16^](https://www.zotero.org/google-docs/?pydpNt) | No;  Requires pre-generated enrichment results from specific tools or in a strict format. | Yes | No | No | Limited choice of algorithms with fixed parameters (e.g., Markov clustering, hierarchical clustering with a fixed cutoff). | Yes;  Selects a single representative pathway based on network connectivity (PageRank, HITS) or statistical significance (max \|NES\| or min *p*-value). | No |
| PAVER[^17^](https://www.zotero.org/google-docs/?SNazee) | No;  Requires three separate inputs: enrichment results, a pathway ID map, and precomputed pathway embeddings. | No | No | Yes | A single hierarchical clustering method | Yes;  Selects a single representative pathway based on the highest cosine similarity to the cluster's average embedding. | No |
| MAPA | Yes;  Accepts a direct list of gene, protein, or metabolite identifiers, automating the enrichment step. | Yes | Yes | Yes | Offers a suite of hierarchical, graph-based, and binary cut clustering algorithms | Yes;  Provides two approaches: (1) LLM-powered interpretation for module naming, and (2) Traditional selection of the most significant pathway. | Yes;  LLM-powered interpretation based on both database knowledge and current PubMed literature |

## Supplementary Note 1. Key limitations of existing pathway similarity metrics.

Multiple algorithms exist for computing pathway similarity, and they can be broadly classified into three categories. First, component-based similarity metrics[^5^](https://www.zotero.org/google-docs/?IxmzBG), such as the Jaccard index, overlap coefficient, Dice Coefficient, and Cohen’s Kappa, quantify the degree of overlap in molecular components (e.g., genes, proteins, or metabolites) between pathway pairs. These methods are simple, interpretable, and applicable across different databases. However, they suffer from key limitations: they are highly sensitive to pathway size, often inflate similarity for large pathways, and disregard descriptive or contextual information[^18^](https://www.zotero.org/google-docs/?RcQdBE).

Second, ontology-based similarity metrics, including the Wang[^19^](https://www.zotero.org/google-docs/?0JPK5f), Resnik[^20^](https://www.zotero.org/google-docs/?JcbeNk), and Lin[^21^](https://www.zotero.org/google-docs/?tvGYvy) algorithms, leverage the structured hierarchy of ontologies such as the Gene Ontology (GO)[^22,23^](https://www.zotero.org/google-docs/?pp4yaz). These algorithms can capture nuanced parent-child relationships and broader biological context[^24^](https://www.zotero.org/google-docs/?6crRa4). However, they are restricted to curated ontologies like GO and cannot be applied to general pathway resources such as KEGG and Reactome. Furthermore, their dependence on static ontology graphs limits their adaptability to emerging biological knowledge and cannot be used across databases.

Third, text-based similarity methods, such as PAVER[^17^](https://www.zotero.org/google-docs/?SPdFCf), use text embeddings to represent pathways and compute pathway similarity. For example, while PAVER can capture semantic relationships and work across different databases, it has notable drawbacks. It relies on precomputed embeddings that may not include updates from the latest pathway databases. Users can utilize the built-in precomputed embeddings, but these may be outdated. Alternatively, users must compute embeddings themselves, which requires technical expertise. This dependency on static embeddings and the complexity of generating updated embeddings limit the practical applicability of PAVER.

## Supplementary Note 2. Existing pathway similarity metrics.

*Component-Based Similarity.* These similarity metrics quantify the overlap between pathway/term components and enable cross-database similarity calculations[^25^](https://www.zotero.org/google-docs/?nYpGjZ).

1. Jaccard Index. The Jaccard Index is defined as:

$J(A, B) = \frac{|A \cap B|}{|A \cup B|}$,

where A and B are the number of molecules annotated to each pathway. This metric ranges from 0 (no shared components) to 1 (identical component sets). It emphasizes proportional overlap but can be biased toward larger pathways.

2. Overlap coefficient. The overlap coefficient is calculated as:

$$O(A, B) = \frac{|A \cap B|}{min(|A|, |B|)}$$

This measure corrects for differences in pathway size by normalizing the intersection over the smaller pathway. It is particularly useful for detecting high similarity between pathways of disparate sizes.

3. Kappa coefficient. The kappa coefficient is to quantify the degree of overlap between pathways beyond what would be expected by random chance.

Given two pathways A and B with n total molecules annotated to them, construct a 2×2 contingency table:

|  | In pathway B | Not in pathway B | Row total |
| --- | --- | --- | --- |
| In pathway A | a | b | a + b |
| Not in pathway A | c | d | c + d |
| Column total | a + c | b + d | a + b + c+ d |

Here, a, b, c, and d represent molecule counts, and n = a + b + c + d.

The observed agreement probability is:$p_{obs} = \frac{a + d}{n}$

The expected random agreement probability under the assumption of independence is:

$$p_{exp}=\frac{(a + b)(a + c)}{n^{2}} +\frac{(c + d)(b+d)}{n^{2}}$$

The final kappa coefficient is defined as: $K(A, B) = \frac{p_{obs}-p_{exp}}{1 - p_{exp}}$

Kappa coefficient ranges from -1 to 1, where: 1 indicates perfect agreement between the two pathways’ component sets. 0 indicates the observed agreement is exactly what would be expected by chance. There is no real agreement, only random overlap. < 0 indicates the observed agreement is worse than what would be expected by chance. This suggests a systematic disagreement between the component sets.

4. Dice coefficient. It is calculated by doubling the number of shared components and dividing by the total number of components in both pathways. The Dice coefficient is defined as:

$D(A, B) = \frac{2*|A \cap B|}{|A| + |B|}$,

where A and B are the sets of molecules annotated to each pathway. This metric ranges from 0 (no common components) to 1 (identical component sets). The Dice coefficient is less sensitive to differences in set sizes than the Jaccard index but can still be influenced when there is a very large discrepancy between the number of components in A and B.

*Ontology-based Similarity.* These metrics quantify semantic similarities between GO terms within the same subontology. The semantic similarity metrics employed in this study include the “Wang” method (based on directed acyclic graph structure) and information content-based methods, as described below[^24,26^](https://www.zotero.org/google-docs/?JDujE1):

The “Wang” method quantifies similarity by considering the graph structure of GO, including the relationships between terms and their ancestors in the ontology. It calculates the semantic contribution of all ancestor terms shared between two GO terms, assigning weights based on proximity in the ontology graph. This method can capture subtle biological relationships not evident through component overlap alone. However, it is limited to GO-based analyses and cannot be applied to pathway databases lacking formal ontology structures (*e.g.*, KEGG or Reactome)[^25^](https://www.zotero.org/google-docs/?nPcGMM).

In IC-based methods, each GO term t is assigned an information content $IC(t)=-logp(t)$, where $p(t)$ is the annotation-based probability of observing term t in the GO corpus (e.g., $p(t)=\frac{k}{N}$with k genes annotated to t and N the total genes annotated in GO). Because GO allows multiple parents, two terms (term a and term b) can share several ancestors; the key ancestor used by IC methods is the most informative common ancestor (MICA, term c), which is the common ancestor with the highest IC. All four methods below differ mainly in how they utilize $IC(a)$, $IC(b)$ and $IC(c)$.

1. The Resnik method is defined as:

$sim(a,b)=IC(c)$.

It rewards pairs whose closest shared ancestor is very specific (high IC).

2. The Lin method normalizes the Resnik’s similarity by the specificity of the query terms themselves,

$sim(a,b)=\frac{IC(c)}{(IC(a)+IC(b))/2}$.

This ratio ranges from 0 to 1, and is high only when both terms are close to their MICA and have comparable specificity.

3. The Rel method refines Lin by penalizing MICAs that are common in the corpus. It multiplies Lin’s similarity by a specificity factor $1-p(c)$,

${sim}_{Rel}(a,b)= {sim}_{Lin}(a,b)*[1-p(c)]$.

The effect is to keep similarities high only when the two terms share an informative and relatively rare ancestor; if the MICA is frequent, the factor shrinks the similarity accordingly.

4. The Jiang method calculates similarity from the perspective of semantic distance. It first defines a distance metric $D(a,b)$ based on the IC of the two terms and their MICA:$D(a,b)=IC(a)+IC(b)-2IC(c)$. This distance value represents the amount of information required to transform one term into the other. The final similarity score is then derived as an inverse function of this distance,

$sim(a,b)=1-min(1,IC(a)+IC(b)-2IC(c))$.

This metric ranges from 0 to 1, where a smaller distance corresponds to higher similarity.

## Supplementary Note 3. Biological connections among GO:0098985, GO:0098978, and hsa04724.

These three pathway identifiers represent closely related aspects of glutamatergic neurotransmission. GO:0098985 (asymmetric, glutamatergic, excitatory synapse) is a specialized subtype of GO:0098978 (glutamatergic synapse), both describing cellular components in the Gene Ontology. The KEGG pathway hsa04724 (glutamatergic synapse) describes the biological processes that occur at these synaptic structures. Despite originating from different databases, all three terms describe complementary aspects of the same biological process, glutamatergic synaptic transmission. This biological coherence underlies the high similarity scores produced by biotext embedding (0.64-0.74) and demonstrates the method’s ability to capture functional relationships across database boundaries.

## Supplementary Note 4. Instruction for human experts to generate the module functional annotation.

**Research Task Description**

As a molecular/biochemical/computational biology researcher, please complete the following analysis task based on the provided biological information (name and description) using your professional expertise and literature search capabilities. Please do not use any large language models or AI tools, but rely on your professional background and literature research to complete this work.

**Research Objectives**

1. Identify the core biological process that describes this module based on the provided pathway information.

2. Analyze functional relationships among genes/proteins, focusing on: shared signaling pathways or molecular mechanisms, synergistic or antagonistic interactions, disease relevance and clinical significance.

3. Avoid isolated functional descriptions: Instead of simply listing individual gene/protein functions, group genes/proteins with similar functions and analyze their collective mechanisms of action.

4. Name the module: Provide a concise and accurate name for this functional module, ensuring it highlights key biological processes, disease associations, or molecular mechanisms while avoiding overly generic terms.

5. Assess confidence score (0.00 - 1.00): This score reflects the degree of overlap or convergence of input genes/proteins and pathways on a single, coherent biological process.

6. Integrate research insights: Ensure that the generated summary reflects findings from your literature research.

**Suggested Research Methods**

• Literature Search: Use databases such as PubMed, Web of Science for relevant literature

• Pathway Databases: Consult KEGG, Reactome, GO databases to validate pathway information

Final Output Requirements

Please provide your analysis as below:

Module name: The name of the module.

Summary: A detailed, literature-integrated explanation of the module's function and relevant biological processes.

Confidence score: A value between 0.00 and 1.00.

**Module Analysis Data**

{Module_id}

{pathway_id}:{pathway name and description}

{**Your summary**}

## Supplementary Note 5. Explanation for differences between ground truth and clustering result generated by louvain algorithm (similarity cutoff: 0.55)

**Difference1: The hsa04210 (Apoptosis) pathway failed to cluster with pathways in the expected Functional_module_10**.

In the curated dataset, hsa04210 (Apoptosis) was expected to cluster with pathways in the MAPK signaling module. This is because hsa04010 (MAPK signaling pathway) and hsa04210 are documented in the KEGG database as related pathways, since MAPK signaling is a key upstream regulator of apoptosis. Typical members of MAPKs, such as C-Jun N-terminal kinase (JNK), p38 MAPK, and ERK1/2, can mediate both pro-apoptotic and anti-apoptotic processes, balancing cell survival and death[^27^](https://www.zotero.org/google-docs/?b6KdgR). However, our biotext embedding generated only moderate similarities (0.33-0.52) between hsa04210 and other pathways in this module. When we applied a similarity cutoff of 0.55, hsa04210 became a singleton, separating it from the other pathways. These moderate similarities occurred because the pathway descriptions provided by the databases hardly refer to the association between apoptosis and MAPK, instead putting emphasis on cell survival-related processes. This discrepancy demonstrates that MAPA’s similarity threshold provides users with analytical flexibility. At a higher cutoff (e.g., 0.55), we obtain specific functional modules focused on survival, while lower thresholds might capture broader functional themes encompassing general cell fate control. This allows researchers to tailor their analysis to match their specific research questions.

**Difference2: hsa00190 (Oxidative phosphorylation) failed to cluster with its expected Functional_module_7.**

The hsa00190 pathway was expected to cluster with GO:0009055 (electron transfer activity) and GO:0045251 (electron transfer flavoprotein complex). These processes are highly related, as they are functionally interdependent parts of cellular energy production. Specifically, electron transfer activity and the ETF complex directly power oxidative phosphorylation by moving electrons through the respiratory chain to create the proton gradient for ATP synthesis[^28^](https://www.zotero.org/google-docs/?PXGhMG).

Despite this functional link, hsa00190 failed to cluster with the two GO terms because it only achieved a moderate similarity score of approximately 0.4 with each. We hypothesize this low similarity is due to the brevity of the pathway’s description (Oxidative phosphorylation), which provides insufficient semantic context for the text embedding model. This hypothesis is supported by observing other terms in the dataset with similarly moderate scores (~0.43). These terms typically fall into two categories: One is those with very short descriptions (e.g., hsa00062, “Fatty acid elongation”). The other one is those that share superficial keywords like “phosphorylation,” leading to vocabulary overlap without a highly functional connection (e.g., GO:0004672 (protein kinase activity)). These results suggest that the performance of the biotext embedding similarity might be influenced by text length and simple vocabulary overlap when calculating similarities between biological concepts.

## Supplementary Note 6. Prompt engineering.

**Prompt design for literature reranking**

**System Message:**

You are an AI tasked with identifying the most relevant and valuable articles for the given module.

**User Message Template (example fields shown as placeholders):**

The module is defined by the following pathways: **{pathways}**.

The module also focuses on the following molecules: **{molecules}**.

Below is an abstract or chunk of text. Please:

Rank its relevance to the module in terms of how well it aligns with the pathways and molecules.

Remove unrelated information such as author names, affiliations, and non-relevant metadata.

Return the cleaned text and assign it a relevance score (from 0 to 1, where 1 is highly relevant).

**{chunk}**

Please respond in the following JSON format:

{

"relevance_score": <score>,

"cleaned_text": "<cleaned text>"

}

**Prompt design for single-omics functional module annotation**

\## **Task Description**

You are an AI tasked with generating a **biological module name, a research summary, and a confidence score** based on the following information.

\## **Your Task**

1. **Identify the core biological process** that describes this module.

2. **Analyze functional relationships** among {query_molecules}, considering:

- Shared pathways or mechanisms

- Synergistic/antagonistic interactions

- Disease relevance

3. **Avoid listing isolated {query_product} functions**; instead, group {query_product}s with similar functions and analyze their collective roles.

4. **Name the module concisely**, ensuring it highlights a key biological process, disease association, or molecular mechanism rather than a generic term.

5. Assign a **confidence score (0.00 - 1.00)** This score reflects how strongly the input {query_molecules} and pathways overlap or converge on a single, coherent biological process.

For instance, if the pathways are highly related (e.g., serine-type endopeptidase inhibitor activity and endopeptidase inhibitor activity), the score should be high (close to 1.00).

If the pathways are largely unrelated (e.g., negative regulation of inflammatory response and camera-type eye development), the score should be low (close to 0.00).

Consider whether these pathways genuinely point to one biological theme, whether the {query_molecules} are meaningfully involved in those pathways, and whether literature or research supports a functional connection.

6. **Incorporate research insights**, ensuring the generated summary reflects findings from the provided literature (if provided).

\## **Example Input**

The module is defined by the following pathways:

Transmembrane receptor protein kinase activity

(Catalysis of the reaction: ATP + a protein tyrosine = ADP + protein tyrosine phosphate.)

Transmembrane receptor protein tyrosine kinase activity

(Combining with a signal and transmitting the signal from one side of the membrane to the other to initiate a change in cell activity.)

Protein tyrosine kinase activity

(Catalysis of the reaction: a protein + ATP = a phosphoprotein + ADP.)

The module also focuses on the following genes and/or proteins:

transforming growth factor beta receptor 2, insulin like growth factor 1 receptor, EPH receptor A2, epidermal growth factor receptor, platelet derived growth factor receptor alpha, erb-b2 receptor tyrosine kinase 2, ret proto-oncogene, fms related receptor tyrosine kinase 4.

Below are related articles:

Title: Inhibition of DDR2 reveals kinase-dependent and kinase-independent functions.

Text: Tyrosine kinase signaling plays a crucial role in fibrosis. DDR2, a receptor tyrosine kinase, regulates fibroblast apoptosis and fibrosis progression. Inhibitors like WRG-28 block DDR2 activation, reducing fibrosis severity.

Title: Oncogene-driven receptor tyrosine kinase expression in cancer.

Text: Overexpression of receptor tyrosine kinases like DDR2, RET, and PDGFRA correlates with poor survival in neuroblastoma. The multikinase inhibitor sitravatinib effectively targets DDR2, RET, and PDGFRA, reducing tumor proliferation and metastasis.

\## **Example Output**

{

"module_name": "Receptor Tyrosine Kinase Signaling in Fibrosis and Cancer",

"summary": "This module focuses on the role of receptor tyrosine kinases (RTKs) in fibrosis and cancer, particularly emphasizing pathways related to transmembrane receptor protein kinase activity. Key genes involved include DDR2, PDGFRA, RET, EGFR, and EPH receptors, which regulate cellular signaling in fibroblasts, neuroblastoma, and other disease contexts. The studies highlight DDR2’s central role in fibrosis through collagen autocrine signaling and its involvement in cancer progression. Inhibitors like sitravatinib and WRG-28 have shown promise in targeting DDR2 and other RTKs, affecting tumor growth and fibrosis progression. Additionally, DDR2-mediated PIK3C2α signaling has been implicated in fibroblast function and fibrosis, presenting a potential therapeutic target.",

"confidence_score": "0.95"

}

\## **Actual Input for Generation**

The biological module is defined by the following pathways: {pathway_info}

The module also focuses on the following {query_molecules}: {query_molecule_names}

Below are related articles: {combined_texts}

\## **Final Output**

Please provide your response in **JSON format**, strictly following this structure:

{

"module_name": "Your concise module name here",

"summary": "A detailed, literature-integrated explanation of the module's function, key {query_molecules}, and relevant biological processes.",

"confidence_score": "A value between 0.00 and 1.00, based on {query_molecules}-pathway association and overall functional coherence."

}

When generating your answer, pay attention to the following:

module_name should be concise and descriptive, highlighting the core biological process or disease relevance.

summary should include:

The central biological function or process.

Key {query_molecules} and their roles within the identified pathways.

Insights from the provided articles or research (if provided).

confidence_score should reflect how well the {query_molecules} and pathways associated with each other and support a coherent biological process, with 1.00 indicating very high confidence and 0.00 indicating minimal or no overlap.

**Prompt design for multi-omics functional module annotation**

\## **Task Description**

You are an AI tasked with generating a **module name**, a **function summary**, and a **confidence score** for a multi-omics functional module based on the following information:

- Genes / proteins names

- Metabolites

- Enriched pathways / GO terms (names + short descriptions)

- Related PubMed literature (titles/abstracts or extracted text chunks)

Each information layer may be missing. If pathways/GO terms are missing, do not infer them; you may briefly state that no pathway enrichment terms were provided. If literature is missing or generic, clearly distinguish evidence-backed statements from hypotheses.

Your goal is to generate a coherent biological interpretation of the module, emphasizing **cross-omics consistency** and **mechanistic links**. The summary should read like a concise biological interpretation, not a list of annotations.

---

\## **Your Task**

1. Interpret the biological roles of the genes/proteins, metabolites, and pathway/GO terms.

2. Infer the main biological process or mechanism that best explains the module as a whole.

3. Explain how the different components collectively support this shared theme.

4. Avoid listing isolated component functions or simply restating pathway names.

5. Generate a concise module name that reflects a specific biological process or mechanism.

6. Assign a confidence score (0.00–1.00) based on functional coherence:

- **High (0.8–1.0):** strong convergence across components, with literature support

- **Medium (0.4–0.79):** partial convergence or indirect links

- **Low (0.0–0.39):** weak coherence or insufficient evidence

7. Prefer literature-supported mechanisms when available, and clearly separate supported claims from plausible interpretation.

Keep the summary concise and synthesis-focused. Prioritize the dominant shared mechanism over exhaustive component-level description.

---

\## **Example Input**

The module contains:

**Genes / proteins names:**

- names: acid phosphatase 6, lysophosphatidic acid phosphatase type, inositol polyphosphate-1-phosphatase, inositol-3-phosphate synthase 1, phosphatidylinositol 4-kinase type 2 beta, phosphatidylinositol glycan anchor biosynthesis class S, phosphatidylinositol-5-phosphate 4-kinase type 2 alpha, solute carrier family 44 member 1

**Metabolites:**

- names: D-Glucose 6-phosphate

**Enriched pathways/GO terms (description):**

phospholipid metabolic process (The chemical reactions and pathways involving phospholipids, any lipid containing phosphoric acid as a mono- or diester.)

phospholipid biosynthetic process (The chemical reactions and pathways resulting in the formation of a phospholipid, a lipid containing phosphoric acid as a mono- or diester.)

phosphatidylinositol metabolic process (The chemical reactions and pathways involving phosphatidylinositol, any glycophospholipid in which a sn-glycerol 3-phosphate residue is esterified to the 1-hydroxyl group of 1D-myo-inositol.)

[Additional pathways omitted in this example for brevity.]

**Related articles (PubMed-derived):**

Title: PIP4K2B is mechanoresponsive and controls heterochromatin-driven nuclear softening through UHRF1. (PubMedID:36918565)

Text: Phosphatidylinositol-5-phosphate (PtdIns5P)-4-kinases (PIP4Ks) are stress-regulated phosphoinositide kinases able to phosphorylate PtdIns5P to PtdIns(4,5)P2. Among the three PIP4K isoforms expressed in mammalian cells, PIP4K2B shows prominent nuclear localisation. PIP4K2B protein level strongly decreases in cells growing on soft substrates. Its silencing or pharmacological inhibition reduces the epigenetic regulator UHRF1 and induces changes in nuclear polarity, nuclear envelope tension, and chromatin compaction. This rewiring of nuclear mechanical state drives YAP cytoplasmic retention, impairs its transcriptional activity, and leads to defects in cell spreading and motility. These findings suggest that PIP4K2B links phosphoinositide metabolism to mechanoresponsive nuclear regulation.

[Additional articles omitted in this example for brevity.]

---

\## **Example Output**

{

"module_name": "Phosphatidylinositol and membrane phospholipid metabolism",

"summary": "This module is most consistently explained by a shared role in phosphatidylinositol-centered membrane lipid metabolism and broader glycerophospholipid biosynthesis/remodeling. Several components converge on the inositol–phosphoinositide axis: ISYNA1 links central carbon metabolism to inositol production, making D-glucose 6-phosphate a plausible upstream precursor input for phosphatidylinositol synthesis, while PI4K2B, PIP4K2A, and INPP1 together indicate active interconversion of phosphoinositide species through phosphorylation and dephosphorylation. This core lipid-signaling branch is reinforced by the enriched pathways, which repeatedly point to phospholipid, glycerophospholipid, and phosphatidylinositol metabolic and biosynthetic processes. Additional components broaden the module toward membrane lipid remodeling and utilization: ACP6 is consistent with lysophosphatidic acid/phospholipid turnover, SLC44A1 supports the choline-related branch of phosphatidylcholine metabolism, and PIGS links phosphatidylinositol-derived lipids to GPI-anchor biosynthesis. Together, these layers support a coherent interpretation of the module as a membrane phospholipid metabolic program with a particularly strong phosphoinositide component. The provided literature most directly supports the phosphoinositide branch, showing that PIP4K2B functions as a stress- and mechanoresponsive phosphoinositide kinase that can couple lipid signaling to nuclear mechanical regulation and YAP-related transcriptional control. This literature-backed mechanism strengthens the interpretation that the module is not only involved in structural membrane lipid metabolism but may also contribute to signaling processes responsive to cellular mechanical state. The broader extension of the module to membrane remodeling and lipid biosynthesis is plausible from the combined gene, metabolite, and pathway evidence, although that part is supported more by functional convergence than by the single literature example alone.",

"confidence_score": 0.89

}

Use the Example Input/Output only as a formatting guide. Do not reuse its biological theme unless it is directly supported by the actual input.

---

\## **Actual Input for Generation**

The multi-omics module is defined by the following components.

**Genes / proteins names:**

- names: {GeneNames_vec}

**Metabolites:**

- names: {MetNames_vec}

**Enriched pathways/GO terms (description):**

{combined_pathway_text}

**Related articles (PubMed-derived):**

{combined_texts}

---

\## **Final Output**

When a component (especially pathways/terms) is not provided, keep the summary fully grounded in the available evidence (genes/metabolites/literature).

Please provide your response in **JSON format**, strictly following this structure:

{

"module_name": "Your concise module name here",

"summary": "A coherent biological interpretation of the module, emphasizing cross-omics consistency and mechanistic links",

"confidence_score": "A value between 0.00 and 1.00, reflecting overall functional coherence across omics layers."

}

**Prompt design for meta-module annotation**

\## Task Description

You will be given a meta-module containing multiple functional modules from different tissues. Your goal is to:

1. **Generate a concise, biologically meaningful meta-module name** (3-8 words)

2. **Provide a comprehensive summary** explaining the biological significance of this meta-module.

\## Input Information Format

For each functional module in the meta-module, you will receive:

- **Module Summary**: Functional description of the module

- **Tissue**: The tissue where this module was identified

\## Analysis Guidelines

\### For Meta-module Naming:

- Focus on the **core biological process** or **pathway theme** shared across functional modules

- Consider **tissue specificity** if relevant (e.g., "Cardiac-specific", "Multi-tissue")

- Use standard biological terminology

- Keep it concise but informative

\### For Summary Generation:

- **Identify common biological themes** across all modules in the meta-module

- **Mention key pathways or processes** represented in the meta-module

- **Consider cross-tissue communication** or shared regulatory mechanisms

- **Highlight their biological relevance**

\## Output Format

Respond with a valid JSON object using exactly this format (no markdown, no code blocks, no additional text):

{"meta-module_name": "Your concise meta-module name here", "summary": "A comprehensive biological summary explaining the meta-module's significance, tissue patterns, and potential biological implications"}

Start your response directly with the opening brace { and end with the closing brace }.

\## **Actual Input for Generation**

**Functional modules information in this meta-modules**:

{module_data}

\## Example Analysis Approach

1. Read through all functional module summaries to identify common themes

2. Note tissue distribution

3. Consider biological relationships between tissues

4. Formulate a name that captures the essence of the meta-module

5. Write a summary that explains the biological story this meta-module tells

Please analyze the provided meta-modules and generate the meta-module name and summary following the guidelines above.

## Supplementary Note 7. R version and R packages used for data analysis in this study.

R version 4.5.0 (2025-04-11)

Platform: aarch64-apple-darwin20

Running under: macOS Sequoia 15.6

attached base packages:

[1] stats	graphics grDevices utils	datasets methods	base

other attached packages:

[1] paletteer_1.6.0	ggthemes_5.1.0	AnnotationHub_3.16.0 BiocFileCache_2.16.0 dbplyr_2.5.0	BiocGenerics_0.54.0

[7] generics_0.1.4	data.table_1.17.4	rio_1.2.3	lubridate_1.9.4	forcats_1.0.0	stringr_1.5.1

[13] purrr_1.0.4	readr_2.1.5	tidyr_1.3.1	tibble_3.2.1	tidyverse_2.0.0	mapa_2.1.0

[19] ggplot2_3.5.2	dplyr_1.1.4	masstools_1.0.15	massdataset_1.0.34	magrittr_2.0.3	r4projects_0.1.6

loaded via a namespace (and not attached):

[1] IRanges_2.42.0	R.methodsS3_1.8.2	dichromat_2.0-0.1	koRpus_0.13-8

[5] progress_1.2.3	vsn_3.76.0	Biostrings_2.76.0	vctrs_0.6.5

[9] ggtangle_0.0.6	digest_0.6.37	png_0.1-8	shape_1.4.6.1

[13] proxy_0.4-27	MSnbase_2.34.1	BiocBaseUtils_1.10.0	ggrepel_0.9.6

[17] koRpus.lang.en_0.1-4	parallelly_1.44.0	Rdisop_1.68.0	metid_1.3.1

[21] MASS_7.3-65	reshape2_1.4.4	foreach_1.5.2	httpuv_1.6.16

[25] qvalue_2.40.0	withr_3.0.2	sylly.en_0.1-3	xfun_0.52

[29] ggfun_0.1.8	MetaboCoreUtils_1.16.1	memoise_2.0.1	clusterProfiler_4.16.0

[33] gson_0.1.0	tidytree_0.4.6	lwgeom_0.2-14	GlobalOptions_0.1.2

[37] simplifyEnrichment_2.2.0	pbapply_1.7-2	R.oo_1.27.1	prettyunits_1.2.0

[41] rematch2_2.1.2	KEGGREST_1.48.0	promises_1.3.3	scatterplot3d_0.3-44

[45] httr_1.4.7	globals_0.18.0	rstudioapi_0.17.1	UCSC.utils_1.4.0

[49] units_0.8-7	DOSE_4.2.0	reactome.db_1.92.0	stars_0.6-8

[53] curl_6.2.3	ncdf4_1.24	S4Vectors_0.46.0	ggraph_2.2.1

[57] rtiktoken_0.0.7	polyclip_1.10-7	GenomeInfoDbData_1.2.14	SparseArray_1.8.0

[61] xtable_1.8-4	ade4_1.7-23	doParallel_1.0.17	evaluate_1.0.3

[65] S4Arrays_1.8.0	tmaptools_3.2	preprocessCore_1.70.0	hms_1.1.3

[69] GenomicRanges_1.60.0	ggwordcloud_0.6.2	colorspace_2.1-1	filelock_1.0.3

[73] NLP_0.3-2	readxl_1.4.5	later_1.4.2	viridis_0.6.5

[77] ggtree_3.16.0	lattice_0.22-7	MsCoreUtils_1.20.0	XML_3.99-0.18

[81] cowplot_1.1.3	matrixStats_1.5.0	class_7.3-23	pillar_1.10.2

[85] nlme_3.1-168	iterators_1.0.14	compiler_4.5.0	stringi_1.8.7

[89] sf_1.0-21	SummarizedExperiment_1.38.1 plyr_1.8.9	crayon_1.5.3

[93] abind_1.4-8	gridGraphics_0.5-1	graphlayouts_1.2.2	org.Hs.eg.db_3.21.0

[97] bit_4.6.0	pcaMethods_2.0.0	fastmatch_1.1-6	codetools_0.2-20

[101] slam_0.1-55	e1071_1.7-16	GetoptLong_1.0.5	tm_0.7-16

[105] mime_0.13	MultiAssayExperiment_1.34.0 splines_4.5.0	circlize_0.4.16

[109] Rcpp_1.0.14	cellranger_1.1.0	Rttf2pt1_1.3.12	rbioapi_0.8.2

[113] gridtext_0.1.5	knitr_1.50	blob_1.2.4	clue_0.3-66

[117] BiocVersion_3.21.1	mzR_2.42.0	AnnotationFilter_1.32.0	fs_1.6.6

[121] QFeatures_1.18.0	listenv_0.9.1	mzID_1.46.0	openxlsx_4.2.8

[125] ggplotify_0.1.2	pdftools_3.5.0	Matrix_1.7-3	statmod_1.5.0

[129] tzdb_0.5.0	tweenr_2.0.3	pkgconfig_2.0.3	tools_4.5.0

[133] cachem_1.1.0	RSQLite_2.3.11	viridisLite_0.4.2	rvest_1.0.4

[137] DBI_1.2.3	impute_1.82.0	graphite_1.54.0	fastmap_1.2.0

[141] rmarkdown_2.29	scales_1.4.0	grid_4.5.0	pbmcapply_1.5.1

[145] patchwork_1.3.0	BiocManager_1.30.25	Polychrome_1.5.4	graph_1.86.0

[149] farver_2.1.2	tidygraph_1.3.1	yaml_2.3.10	MatrixGenerics_1.20.0

[153] cli_3.6.5	writexl_1.5.4	stats4_4.5.0	lifecycle_1.0.4

[157] askpass_1.2.1	Biobase_2.68.0	BiocParallel_1.42.0	timechange_0.3.0

[161] gtable_0.3.6	rjson_0.2.23	textstem_0.1.4	parallel_4.5.0

[165] ape_5.8-1	limma_3.64.1	jsonlite_2.0.0	bit64_4.6.0-1

[169] yulab.utils_0.2.0	ReactomePA_1.52.0	zip_2.3.3	metpath_1.0.10

[173] GOSemSim_2.34.0	R.utils_2.13.0	lazyeval_0.2.2	shiny_1.10.0

[177] htmltools_0.5.8.1	affy_1.86.0	enrichplot_1.28.4	sylly_0.1-6

[181] GO.db_3.21.0	rappdirs_0.3.3	glue_1.8.0	factoextra_1.0.7

[185] httr2_1.1.2	XVector_0.48.0	qpdf_1.3.5	treeio_1.32.0

[189] MALDIquant_1.22.3	classInt_0.4-11	gridExtra_2.3	igraph_2.1.4

[193] extrafontdb_1.0	R6_2.6.1	cluster_2.1.8.1	Spectra_1.18.2

[197] stringdist_0.9.15	aplot_0.2.5	GenomeInfoDb_1.44.0	DelayedArray_0.34.1

[201] tidyselect_1.2.1	ProtGenerics_1.40.0	ggforce_0.4.2	xml2_1.3.8

[205] AnnotationDbi_1.70.0	future_1.49.0	KernSmooth_2.23-26	clusterSim_0.51-5

[209] furrr_0.3.1	affyio_1.78.0	fgsea_1.34.0	ComplexHeatmap_2.24.0

[213] RColorBrewer_1.1-3	rlang_1.1.6	extrafont_0.19	remotes_2.5.0

[217] rentrez_1.2.3	simona_1.6.0	PSMatch_1.12.0

# References

[1.](https://www.zotero.org/google-docs/?HSbGP9)	[Carbon, S. *et al.* AmiGO: online access to ontology and annotation data. *Bioinformatics* **25**, 288–289 (2009).](https://www.zotero.org/google-docs/?HSbGP9)

[2.](https://www.zotero.org/google-docs/?HSbGP9)	[Zhang, Y. *et al.* Predicting functions of uncharacterized gene products from microbial communities. *Nat. Biotechnol.* https://doi.org/10.1038/s41587-025-02813-7 (2025) doi:10.1038/s41587-025-02813-7.](https://www.zotero.org/google-docs/?HSbGP9)

[3.](https://www.zotero.org/google-docs/?HSbGP9)	[Scrucca, L., Fraley, C., Murphy, T. B. & Raftery, A. E. *Model-Based Clustering, Classification, and Density Estimation Using Mclust in R*. (Chapman and Hall/CRC, 2023). doi:10.1201/9781003277965.](https://www.zotero.org/google-docs/?HSbGP9)

[4.](https://www.zotero.org/google-docs/?HSbGP9)	[Antonov, M. *et al.* igraph enables fast and robust network analysis across programming languages. (2023).](https://www.zotero.org/google-docs/?HSbGP9)

[5.](https://www.zotero.org/google-docs/?HSbGP9)	[Gu, Z. & Hübschmann, D. SimplifyEnrichment: A Bioconductor Package for Clustering and Visualizing Functional Enrichment Results. *Genomics Proteomics Bioinformatics* **21**, 190–202 (2023).](https://www.zotero.org/google-docs/?HSbGP9)

[6.](https://www.zotero.org/google-docs/?HSbGP9)	[Constantin, A.-E. & Patil, I. ggsignif: R Package for Displaying Significance Brackets for ‘ggplot2’. *PsyArxiv* https://doi.org/10.31234/osf.io/7awm6 (2021) doi:10.31234/osf.io/7awm6.](https://www.zotero.org/google-docs/?HSbGP9)

[7.](https://www.zotero.org/google-docs/?HSbGP9)	[Meissel, K. & Yao, E. S. Using Cliff’s Delta as a Non-Parametric Effect Size Measure: An Accessible Web App and R Tutorial.](https://www.zotero.org/google-docs/?HSbGP9)

[8.](https://www.zotero.org/google-docs/?HSbGP9)	[Torchiano, M. Effsize - A package for efficient effect size computation. (2016) doi:10.5281/ZENODO.1480624.](https://www.zotero.org/google-docs/?HSbGP9)

[9.](https://www.zotero.org/google-docs/?HSbGP9)	[Melville, J. *Uwot: The Uniform Manifold Approximation and Projection (UMAP) Method for Dimensionality Reduction*. (2026).](https://www.zotero.org/google-docs/?HSbGP9)

[10.](https://www.zotero.org/google-docs/?HSbGP9)	[Yang, Y. *et al.* Metformin decelerates aging clock in male monkeys. *Cell* **187**, 6358-6378.e29 (2024).](https://www.zotero.org/google-docs/?HSbGP9)

[11.](https://www.zotero.org/google-docs/?HSbGP9)	[Zhou, G. & Xia, J. OmicsNet: a web-based tool for creation and visual analysis of biological networks in 3D space. *Nucleic Acids Res.* **46**, W514–W522 (2018).](https://www.zotero.org/google-docs/?HSbGP9)

[12.](https://www.zotero.org/google-docs/?HSbGP9)	[Bodein, A., Scott-Boyer, M.-P., Perin, O., Lê Cao, K.-A. & Droit, A. Interpretation of network-based integration from multi-omics longitudinal data. *Nucleic Acids Res.* **50**, e27–e27 (2022).](https://www.zotero.org/google-docs/?HSbGP9)

[13.](https://www.zotero.org/google-docs/?HSbGP9)	[Zhou, G., Ewald, J. & Xia, J. OmicsAnalyst: a comprehensive web-based platform for visual analytics of multi-omics data. *Nucleic Acids Res.* **49**, W476–W482 (2021).](https://www.zotero.org/google-docs/?HSbGP9)

[14.](https://www.zotero.org/google-docs/?HSbGP9)	[Muller, E., Shiryan, I. & Borenstein, E. Multi-omic integration of microbiome data for identifying disease-associated modules. *Nat. Commun.* **15**, 2621 (2024).](https://www.zotero.org/google-docs/?HSbGP9)

[15.](https://www.zotero.org/google-docs/?HSbGP9)	[Yu, G. *Enrichplot: Visualization of Functional Enrichment Result*. (2025). doi:10.18129/B9.bioc.enrichplot.](https://www.zotero.org/google-docs/?HSbGP9)

[16.](https://www.zotero.org/google-docs/?HSbGP9)	[Kerseviciute, I. & Gordevicius, J. *aPEAR* : an R package for autonomous visualization of pathway enrichment networks. *Bioinformatics* **39**, btad672 (2023).](https://www.zotero.org/google-docs/?HSbGP9)

[17.](https://www.zotero.org/google-docs/?HSbGP9)	[Ryan V, W. G. *et al.* Interpreting and visualizing pathway analyses using embedding representations with PAVER. *Bioinformation* **20**, 700–704 (2024).](https://www.zotero.org/google-docs/?HSbGP9)

[18.](https://www.zotero.org/google-docs/?HSbGP9)	[Ballouz, S., Pavlidis, P. & Gillis, J. Using predictive specificity to determine when gene set analysis is biologically meaningful. *Nucleic Acids Res.* gkw957 (2016) doi:10.1093/nar/gkw957.](https://www.zotero.org/google-docs/?HSbGP9)

[19.](https://www.zotero.org/google-docs/?HSbGP9)	[Wang, J. Z., Du, Z., Payattakool, R., Yu, P. S. & Chen, C.-F. A new method to measure the semantic similarity of GO terms. *Bioinforma. Oxf. Engl.* **23**, 1274–1281 (2007).](https://www.zotero.org/google-docs/?HSbGP9)

[20.](https://www.zotero.org/google-docs/?HSbGP9)	[Resnik, P. Semantic Similarity in a Taxonomy: An Information-Based Measure and its Application to Problems of Ambiguity in Natural Language. *J. Artif. Intell. Res.* **11**, 95–130 (1999).](https://www.zotero.org/google-docs/?HSbGP9)

[21.](https://www.zotero.org/google-docs/?HSbGP9)	[Lin, D. An Information-Theoretic Definition of Similarity. in *Proceedings of the Fifteenth International Conference on Machine Learning* 296–304 (Morgan Kaufmann Publishers Inc., San Francisco, CA, USA, 1998).](https://www.zotero.org/google-docs/?HSbGP9)

[22.](https://www.zotero.org/google-docs/?HSbGP9)	[Ashburner, M. *et al.* Gene Ontology: tool for the unification of biology. *Nat. Genet.* **25**, 25–29 (2000).](https://www.zotero.org/google-docs/?HSbGP9)

[23.](https://www.zotero.org/google-docs/?HSbGP9)	[The Gene Ontology Consortium. The Gene Ontology knowledgebase in 2026. *Nucleic Acids Res.* **54**, D1779–D1792 (2026).](https://www.zotero.org/google-docs/?HSbGP9)

[24.](https://www.zotero.org/google-docs/?HSbGP9)	[Yu, G. *et al.* GOSemSim: an R package for measuring semantic similarity among GO terms and gene products. *Bioinformatics* **26**, 976–978 (2010).](https://www.zotero.org/google-docs/?HSbGP9)

[25.](https://www.zotero.org/google-docs/?HSbGP9)	[Mazandu, G. K., Chimusa, E. R. & Mulder, N. J. Gene Ontology semantic similarity tools: survey on features and challenges for biological knowledge discovery. *Brief. Bioinform.* **18**, 886–901 (2017).](https://www.zotero.org/google-docs/?HSbGP9)

[26.](https://www.zotero.org/google-docs/?HSbGP9)	[Yu, G. Gene Ontology Semantic Similarity Analysis Using GOSemSim. *Methods Mol. Biol. Clifton NJ* **2117**, 207–215 (2020).](https://www.zotero.org/google-docs/?HSbGP9)

[27.](https://www.zotero.org/google-docs/?HSbGP9)	[Yue, J. & López, J. M. Understanding MAPK Signaling Pathways in Apoptosis. *Int. J. Mol. Sci.* **21**, 2346 (2020).](https://www.zotero.org/google-docs/?HSbGP9)

[28.](https://www.zotero.org/google-docs/?HSbGP9)	[Ahmad, M., Wolberg, A. & Kahwaji, C. I. Biochemistry, Electron Transport Chain. in *StatPearls* (StatPearls Publishing, Treasure Island (FL), 2025).](https://www.zotero.org/google-docs/?HSbGP9)
